# Molecular and expression analyses indicate the role of fusion transcripts in mediating abiotic stress responses in chickpea

**DOI:** 10.3389/fpls.2025.1677098

**Published:** 2025-10-31

**Authors:** Fiza Hamid, Shafaque Zahra, Shailesh Kumar

**Affiliations:** ^1^ Bioinformatics Lab, BRIC-National Institute of Plant Genome Research, New Delhi, India; ^2^ Department of Pathology, School of Medicine, University of Virginia, Charlottesville, VA, United States

**Keywords:** abiotic stress, *Cicer arietinum*, fusion transcripts, trans-splicing, transcriptome complexity

## Abstract

Understanding the transcriptome diversity is essential for deciphering the transcriptional level regulation. High-throughput sequencing technologies have facilitated the detection of fusion transcripts (FTs), which are chimeric mRNA molecules derived from gene fusions due to chromosomal rearrangements or via the splicing machinery at the RNA level. In this study, we investigated the transcriptome complexity in *Cicer arietinum* resulting from fusion events using high-throughput RNA-Seq datasets from five tissues, i.e., stem, leaves, buds, flowers, and pods, and two abiotic stress conditions, i.e., drought and salinity. Of the 328 unique FTs identified, 69% exhibited the presence of canonical splice sites at their junction, indicating their generation via trans-splicing. Functional annotation and enrichment analyses of fusion partners suggested that these transcripts may expand functional diversity. A total of 10 FTs were validated via RT-PCR followed by Sanger sequencing, which are the first FTs described in the important legume chickpea. Expression analysis of fusion transcripts across various tissues and under abiotic stress conditions revealed evidence of context-dependent regulation. Furthermore, 120 fusion gene pairs were found to be conserved across 17 chickpea genotypes, highlighting their potential biological significance and stability within the species. Overall, these findings suggest that fusion transcripts may contribute to regulatory mechanisms underlying abiotic stress responses in chickpea.

## 1 Introduction

Mature mRNA molecules are conventionally formed through transcription and post-transcriptional modifications, where introns are excised and stability is enhanced. Traditionally, mRNA was thought to originate solely from the alternative splicing of a single gene. However, advancements in sequencing technologies have uncovered the existence of novel transcripts, such as fusion transcripts (FTs), which arise by the joining of mRNA molecules derived from different genes (Gingeras, 2009; Gupta et al., 2018). FTs can be generated at the DNA level through genomic rearrangements such as translocation, deletion, duplication, or inversion and give rise to a fused gene (Li et al., 2009a; Annala et al., 2013); or at the RNA level through trans-splicing (Sutton and Boothroyd, 1986) or read-through transcription (Varley et al., 2014), resulting in an increase in transcriptome complexity without a corresponding increase in the gene number. While fusion transcripts are found in both unicellular and multicellular organisms, they were previously considered rare in nature and occasionally dismissed as transcriptional artifacts. Up to now, extensive research has characterized the cellular functions of these transcripts in cancer (Latysheva and Babu, 2016; Dupain et al., 2019). Recent studies indicate that in addition to their role in oncogenesis, FTs have also been reported under normal physiological conditions (Babiceanu et al., 2016; Chwalenia et al., 2017), and may act as potential regulators of their parental mRNA (Mukherjee et al., 2021). However, their presence and functional significance in plants remain largely unexplored.

In past decades, efficient big data analysis has facilitated the discovery of fusion transcripts in many plants, such as *Trifolium pratense* (Chao et al., 2018)*, Camellia sinensis* (Qiao et al., 2019), *Oryza sativa* (Zhang et al., 2010), *Arabidopsis thaliana* (Singh et al., 2019), *Brassica rapa* (Tan et al., 2019), and *Zea mays* (Zhou et al., 2022). These plant-specific fusion transcripts add transcriptome complexity and introduce novel functions, which may be related to stress responses (Zhou et al., 2022), metabolic pathways (Hagel and Facchini, 2017), or phenotypic traits (Chen et al., 2017). For instance, maize exhibits various fusion events in response to viral infection, involving proteins like nodulin, flavone synthase, and cation-transporting ATPase (Zhou et al., 2022). In tomatoes, the fusion transcript *PFP-LeT6* is involved in leaf patterning (Kim et al., 2001), while in rice, the *GN2* chimeric gene controls plant height, heading date, and grain number (Chen et al., 2017). In *Arabidopsis thaliana*, a protein derived from the fusion of glutamine synthase and nodulin domains regulates root morphogenesis and flagellin-triggered signaling (Doskočilová et al., 2011). Studies across species also suggest that fusion events like domain shuffling and DNA fusion enhance the catalytic efficiency of an enzyme (Farrow et al., 2015; Li et al., 2016; Hagel and Facchini, 2017). These reports suggest that fusion events result in novel sequences with novel functions either as non-coding RNA or fusion proteins (Winzer et al., 2015; Qin et al., 2017).

Chickpea (*Cicer arietinum L*.) ranks as the third most cultivated legume crop globally, following common bean and pea (Nathawat et al., 2024). Cultivated in over 50 countries, the Indian subcontinent leads in production, contributing approximately 70% of the global output (Koul et al., 2022). In addition to its economic significance, chickpeas are highly esteemed for their exceptional nutritional content, particularly their rich protein and carbohydrate content. Currently, chickpeas are grown on 15 million hectares worldwide, producing 15.9 million tons (FAO, 2023). However, this yield remains significantly below the crop’s potential under optimal conditions. This gap is largely attributed to various biotic and abiotic stresses that hinder productivity. Abiotic stresses, such as salinity and drought, are key factors contributing to yield losses. Research has shown that chickpeas are particularly sensitive to salinity compared to other crops, making it a critical factor limiting yields (Flowers et al., 2010; Turner et al., 2013). The growing availability of high-throughput transcriptomic data offers valuable insights for tackling these stress-related challenges.

Despite advancements in sequencing technologies, the exploration of transcriptome diversity in chickpea, particularly due to fusion events, remains limited. A meta-analysis in *Arabidopsis thaliana*, *Cicer arietinum*, and *Oryza sativa* showed that the majority of fusion transcripts (~74%) were uniquely detected in only one transcriptomic sample (Chitkara et al., 2024). This lack of recurrence raises concerns about the biological reliability and reproducibility of these events. One possible explanation could be the inherent variability introduced by such a large and heterogeneous dataset. Alternatively, many of these uniquely detected fusion transcripts might represent random artifacts rather than true biological events. Moreover, the validation rate was reported to be generally low in *Arabidopsis thaliana* and *Oryza sativa*, which could be attributed to high false-positive prediction rates or the inherently low expression level of fusion transcripts. Notably, no experimental validation was performed for the fusion transcripts identified in *Cicer arietinum*.

Therefore, there is a compelling need for more targeted investigations under well-defined experimental conditions that allow biological replication. In the current study, we identified, annotated, and experimentally validated the fusion transcripts by utilizing in-house generated RNA-Seq datasets derived from specific abiotic stress conditions and tissue types, enabling the identification of high-confidence fusion transcripts by applying stringent filtering criteria. This enhances the reproducibility, which in turn strengthens the confidence in these fusion events as biologically relevant and potentially regulatory molecules, rather than technical noise. Further, stress-responsive and conserved fusion transcripts of chickpea were confirmed using a combination of second- and third-generation RNA-Seq datasets, along with experimental validation. To explore the features of fusion transcripts, we investigated their coding potential and the potential mechanism of formation. We predicted that 58% of fusion transcripts are non-coding in nature and 69% might arise via trans-splicing. While these findings suggest that non-coding fusion transcripts could play regulatory roles, their functional implications remain speculative and require further experimental validation. Additionally, a benchmarking analysis was performed to evaluate the performance of different fusion detection tools, enabling the identification of the most effective methods for accurate fusion detection in Chickpea. This work provides deeper biological interpretation and functional insights into fusion transcript dynamics in Chickpea.

## 2 Materials and methods

### 2.1 Plant material, growth conditions, and stress treatment

Chickpea (Genotype ICC4958) seeds were cultivated by using the established protocol (Garg et al., 2010). Seedlings were grown in plastic pots filled with a sterilized mix of agro-peat and vermiculite in a 1:1 ratio, maintained at 22 °C with a 14-hour light cycle in a controlled growth chamber. Samples were collected at various developmental stages, including stems and leaves from the seedling stage and buds, flowers, and pods during the flowering stage. To induce drought stress, 21-day-old seedlings were removed from the pots and placed on tissue paper for five hours. For salinity stress, seedlings were immersed in a beaker filled with a 150 mM NaCl solution at 22 °C. Whole seedlings were collected after 5 hours of treatment and from three independent biological replicates. These samples were immediately flash-frozen in liquid nitrogen and stored at -80 °C until further analysis.

### 2.2 Total RNA isolation, cDNA library preparation, and sequencing

Total RNA was extracted from 100 mg of tissue using the RNeasy Plant Mini Kit (Qiagen). The quantity and quality of RNA were assessed using a NanoDrop Spectrophotometer (NanoDrop Technologies) and an Agilent Bioanalyzer. Only samples with a 260/280 ratio between 1.9 and 2.1, a 260/230 ratio between 2.0 and 2.4, and RNA Integrity Number (RIN) values greater than 7 were selected for Illumina sequencing. A total of 26 paired-end RNA-Seq libraries were prepared and sequenced. The quality of the reads was assessed with FastQC (https://www.bioinformatics.babraham.ac.uk/projects/fastqc/), and adapter trimming and filtering of low-quality reads were performed using fastp (Chen et al., 2018). High-quality reads were then aligned to the reference genome (ASM33114v1) using HISAT2-2.2.1 (Kim et al., 2019).

### 2.3 Computational prediction of fusion transcripts and their coding potential

Fusion transcripts were predicted using three different fusion detection tools, viz., FusionMap (Ge et al., 2011), STAR-Fusion (Haas et al., 2019), and MapSplice (Wang et al., 2010). These tools have different fusion detection strategies and filtering criteria, which help in identifying a broad range of fusion events. These tools were selected on the basis of available benchmarking publications, where STAR-Fusion was ranked as the best tool in terms of its high sensitivity, accuracy, and execution time (Haas et al., 2019); and MapSplice and FusionMap show good sensitivity for fusion detection (Kumar et al., 2016). To assess the coding potential of fusion transcripts, we used CNIT (Guo et al., 2019), CPAT (Wang et al., 2013), and PlncPRO (Singh et al., 2017). CNIT uses a support vector machine (SVM) model to predict coding potential based on intrinsic sequence properties. CPAT uses a linear regression model to distinguish between coding and non-coding transcripts. PlncPRO employs a random forest classifier that integrates protein homology, sequence-based features, and 3-mer frequency patterns to differentiate coding transcripts from long non-coding RNAs (lncRNAs). Fusion transcripts were predicted as protein-coding or lncRNA depending on the consensus prediction of at least two of the three tools.

### 2.4 Gene ontology and KEGG pathway analysis

For all identified fusion transcripts, gene ontology (GO) and KEGG pathway analyses were performed using the DAVID web server (Sherman et al., 2022) with the DAVID knowledgebase v2024q2 (released on July 5, 2024), applying a significance cutoff of *p* < 0.05. These analyses help to identify biological processes, cellular components, and pathways potentially impacted by fusion events, shedding light on their functional relevance.

### 2.5 Expression analysis of fusion transcripts

The expression level of genes involved in fusion formation was analyzed based on Transcripts Per Million (TPM) using StringTie (Pertea et al., 2016) with the default parameters. To study the impact of fusion formation on the expression of their parental genes, we categorized the samples into two groups for each fusion transcript: (i) fusion-present (F/P) samples in which the corresponding fusion transcript was detected, and (ii) fusion-absent (F/A) samples lacking the respective fusion. The expression levels of each parental gene were compared between each sample of the two groups. Genes exhibiting ≥2-fold change in expression in F/P samples compared to F/A samples were considered upregulated or downregulated.

### 2.6 Validation using long-read RNA-Seq data

For further validation of fusion transcripts, PacBio long-read RNA-Seq data (PRJNA613159, Jain et al., 2022) for *Cicer arietinum* (ICC4958) were downloaded from the NCBI Sequence Read Archive (SRA). The 200 bp junction sequences of the identified fusion transcripts, comprising 100 bp upstream of the 5′ parental gene breakpoint and 100 bp downstream of the 3′ parental gene breakpoint, were searched against the long-read RNA-Seq data using BLASTn. Hits with greater than 80% sequence identity and alignment length exceeding 150 bp were considered significant. This approach confirmed the presence of predicted fusion transcripts within the long-read data, enhancing confidence in the identified fusion events. Additionally, it offered insights into the putative length of the fusion transcripts.

### 2.7 Identification of conserved fusion events

To identify intra-specific conserved fusions, 103 raw transcriptome data files from 17 *Cicer arietinum* genotypes were downloaded from the NCBI-SRA (https://www.ncbi.nlm.nih.gov/sra). A list of RNA-Seq samples from different genotypes used in this study is listed in **Supplementary Table S8**. Fusion events were then identified across all genotypes using three fusion detection tools. A binary analysis was conducted to determine the presence or absence of each fusion gene pair in the respective genotypes. For inter-specific conserved fusion detection, fusion genes previously reported in *Arabidopsis thaliana* (via AtFusionDB: http://www.nipgr.ac.in/AtFusionDB) were utilized to identify their homologous fusion gene pairs in *Cicer arietinum* using the OrthoFinder tool.

### 2.8 Experimental validation of fusion transcripts

To validate fusion transcripts, primers were designed for the fusion transcript sequences taken from the genome, 200 bp upstream and 200 bp downstream of the breakpoint, using the OligoCalc (Kibbe, 2007) and primer BLAST. All primers used for validation are listed in **Supplementary Table S9**. The cDNA synthesis was carried out with 2µg of RNA using the Verso cDNA synthesis kit (ThermoScientific™). Following RT-PCR and gel electrophoresis, DNA bands were extracted and purified using the GenElute™ Gel Extraction kit (Sigma-Aldrich) and sent for Sanger sequencing at the NIPGR DNA sequencing facility. To quantify the expression of fusion transcripts under drought and salt stress, and across different tissues, quantitative Real-Time PCR was performed. EF1-α was used as an endogenous control gene in this experiment. The real-time PCR reaction mix (10 µl) consisted of 5 µl of 2X SYBR Green Master Mix (Applied Biosystems™), 10 µM of each primer, and 100 ng of cDNA template. PCR amplification was performed using an Applied Biosystems™ qPCR system with thermal cycling conditions of initial denaturation at 95 °C for 2 minutes, followed by 40 cycles of 95 °C for 15 seconds and 60 °C for 1 minute. Each PCR reaction included three biological replicates and three technical replicates. To ensure primer specificity, primer amplification efficiency and melt-curve analyses were performed (**Supplementary Figure S6**). The relative expression levels of fusion transcripts were calculated by using the 2^-ΔΔCt method (Livak and Schmittgen, 2001). The experimental data were presented as the mean with standard deviation (mean ± SD) derived from three independent biological replicates and three technical replicates. Statistical analysis was conducted by comparing the means of control and stressed plants using one-way analysis of variance (ANOVA) followed by Student’s t-test, with a significance level set at *P* < 0.05. *P*-values less than 0.05 were considered statistically significant.

### 2.9 Benchmark analysis of fusion detection tools

To benchmark the performance of various fusion detection tools for *Cicer*, we evaluated STAR-Fusion (Haas et al., 2019), SQUID (Ma et al., 2018), MapSplice (Wang et al., 2010), Tophat-Fusion (Kim and Salzberg, 2011), and FusionMap (Ge et al., 2011). The performance of each tool was assessed using both a public dataset (PRJNA288321, Garg et al., 2016) and our in-house dataset. For both datasets, we then searched the validated fusion transcripts (true fusions) in the results generated by each tool. The accuracy of fusion prediction was calculated following the method described by Kumar et al. (2016) (Kumar et al., 2016), as given below.


$$Sensitivity\;\left(\%\right)=\left(\frac{TP}{TF}\right)*100$$

$$Precision\;\left(\%\right)=\frac{TP}{TP+FP}*100$$

F measure=2∗(S∗P)/(S/P)

S: Sensitivity, P: Precision, TP: True positive, TF: Total fusions, FP: False positive

## 3 Results

### 3.1 Identification of fusion transcripts in *Cicer arietinum*

**Figure 1:**
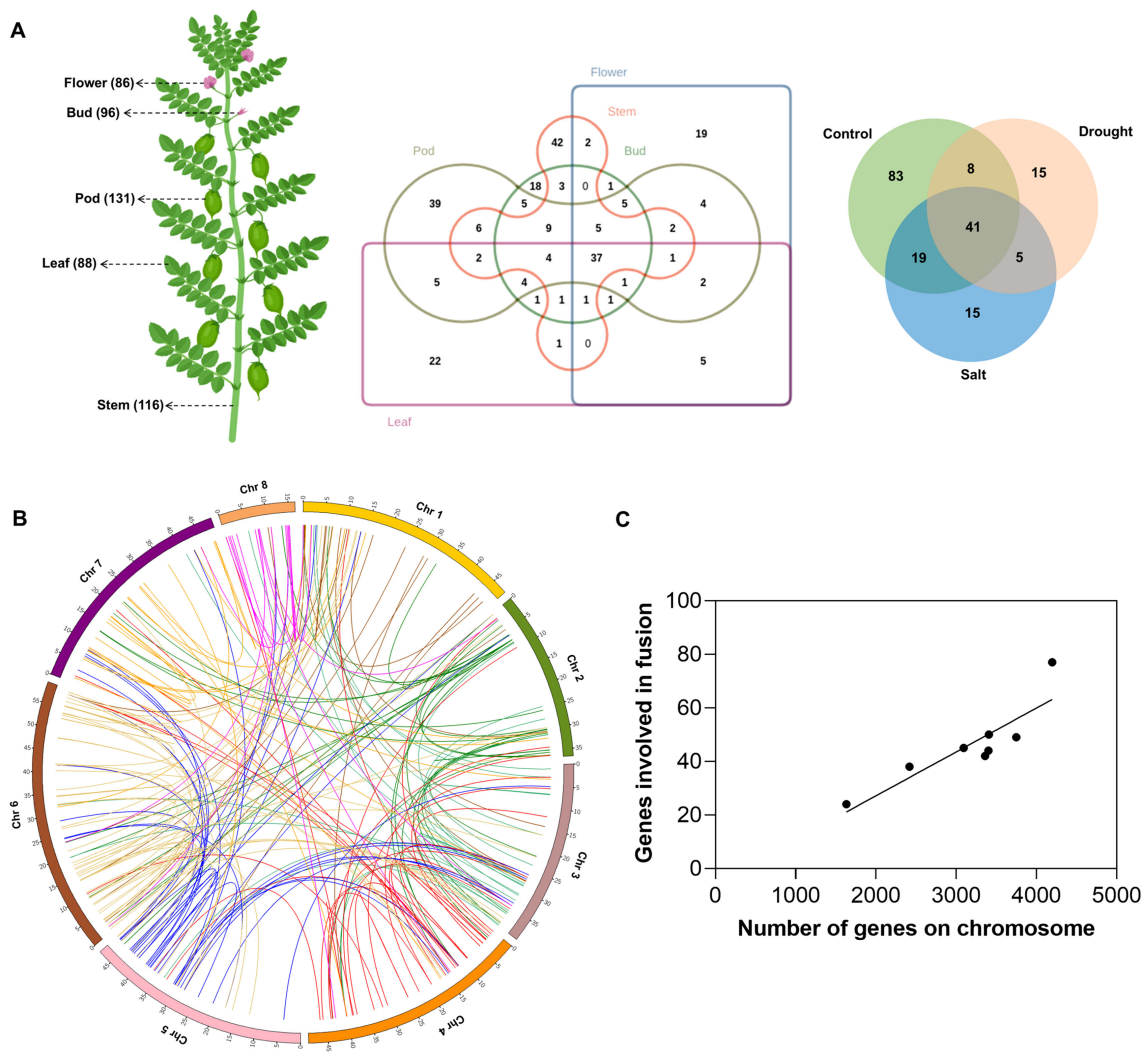
Distribution of fusion transcripts in *Cicer arietinum*, **(A)** The tissues included in this study and the number of fusions detected are indicated within parentheses following the tissue name. Venn diagram showing the overlap in fusion transcripts across different tissues and stress conditions, **(B)** Circos plot depicting the chromosomal distribution of parental genes involved in fusion events, with each connecting link representing a fusion event, **(C)** Correlation between the total number of genes per chromosome and the number of genes involved in fusion formation on that chromosome.

To comprehensively investigate fusion events within the chickpea transcriptome, high-throughput paired-end RNA-Seq data of 150 base pairs (bp) read length were generated using the Illumina sequencing platform. The samples were sequenced from poly(A)-enriched RNAs extracted from five distinct organs, viz., buds, leaves, pods, flowers, and stem (**Figure 1A**), along with two abiotic stress conditions (i.e., drought and salinity). After adapter trimming and quality-check, high-quality reads were mapped onto the CDC Frontier Genome (ASM33114v1) (Varshney et al., 2013) using HISAT2 (v2.2.1) (Kim et al., 2019), and it was found that >95% of the reads mapped onto the genome from each paired-end RNA-Seq sample (**Supplementary Table S1**). In total, 122.47 GB of data was obtained from all samples, representing about 230-fold of the chickpea genome size, and around 852.8 million high-quality reads were generated.

FusionMap (Ge et al., 2011), STAR-Fusion (Haas et al., 2019), and MapSplice (Wang et al., 2010) tools were employed for genome-wide identification of fusion transcripts in 26 RNA-Seq datasets of different tissues, and two abiotic stress conditions (**Supplementary Table S1**). The number of overlapping fusion transcripts detected between different tissues and stress samples is shown in **Figure 1A**. A total of 496, 721, and 533 fusion transcripts were identified by FusionMap (Ge et al., 2011), STAR-Fusion (Haas et al., 2019), and MapSplice (Wang et al., 2010), respectively (**Supplementary Table S2**). However, the number of unique fusion transcripts identified is 95, 109, and 140, respectively, resulting in 328 unique fusions (**Supplementary Table S3**) derived from 269 unique parental gene pairs. In total, 423 genes were involved in fusion formation, accounting for 1.4% (423/30,344) of the total annotated genes in *Cicer arietinum*. This proportion is similar to the percentage of genes involved in fusion events in other plant species, such as *Arabidopsis* (1%), soybean (1.7%), and rice (2.7%) (Cong et al., 2024). To verify the reliability of the predicted fusion transcripts, we randomly selected and validated 10 fusion transcripts in multiple biological replicates by PCR and Sanger sequencing (**Supplementary Figure S1**). The experimentally validated junction sequences precisely matched the in-silico predicted breakpoint sites, confirming the accuracy of the fusion detection approach.

### 3.2 Features of fusion transcripts in *Cicer arietinum*

The prevalence of interchromosomal fusions (76%) as compared to intrachromosomal fusions (24%) in *Cicer arietinum* (**Figure 1B**) indicates that the formation of fusion transcripts is not strictly governed by the linear proximity of parental genes. It suggests that, beyond the linear arrangement of genes, spatial organization may play a role in facilitating fusion events in chickpea and thus underscores the value of further investigation into the spatial organization of the genome. A positive correlation between the number of genes participating in fusion events located at a particular chromosome and the total number of genes on that chromosome suggests the lack of chromosome-level preference in fusion formation (**Figure 1C**). Out of the 423 genes involved in fusion formation, 306 participate in only one unique fusion event, while 70 genes form fusions with two different partner genes. This indicates that fusion events are highly specific, with individual genes preferentially associating with particular partners rather than engaging in multiple fusion events. However, the mechanisms behind partner selection in fusion events remain unknown.

**Figure 2:**
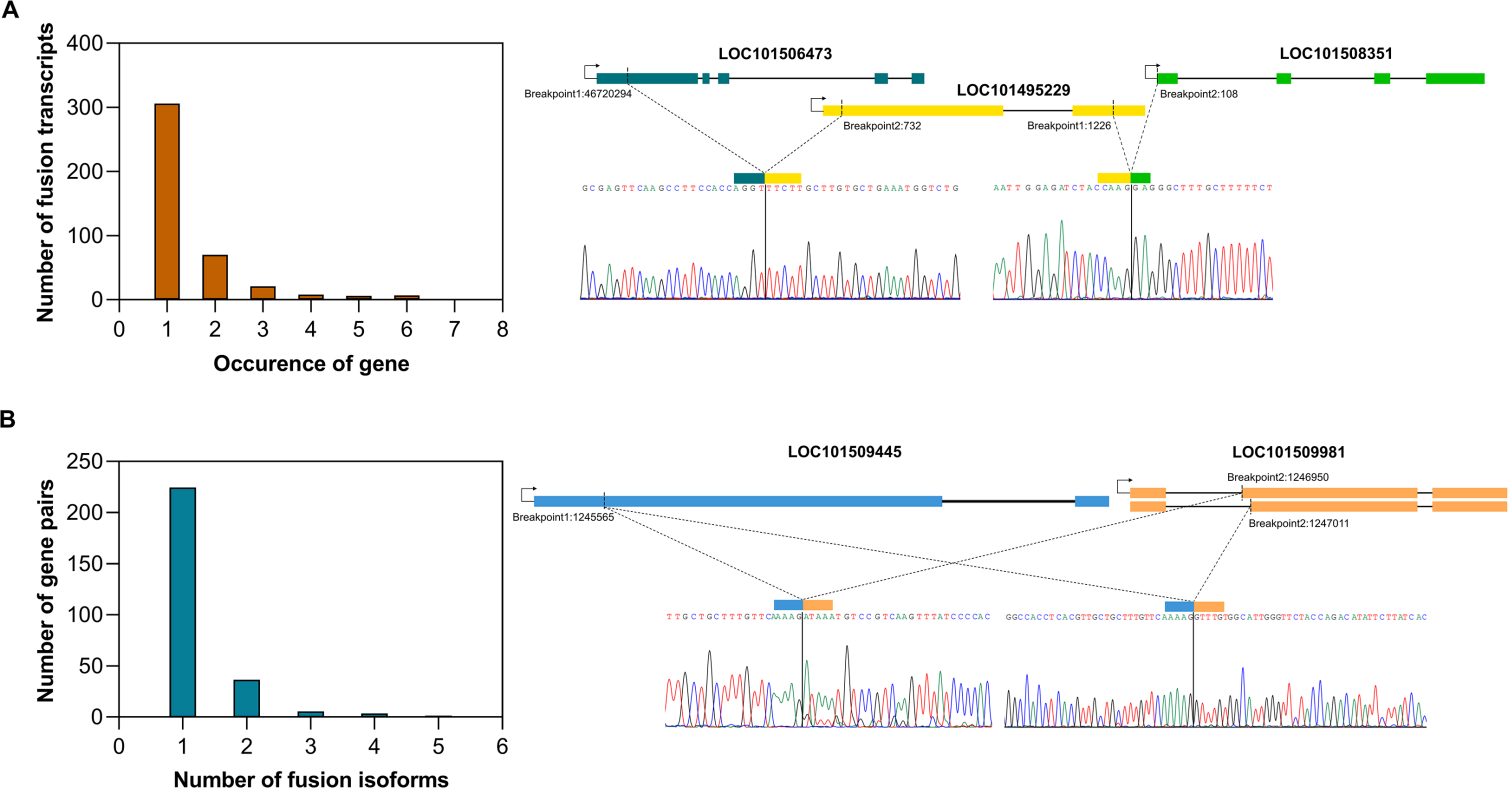
Specificity of fusion events, **(A)** Bar plot showing the frequency of involvement of a gene in multiple fusion events. An example of a gene involved in multiple fusion events is highlighted, **(B)** Bar plot showing the number of isoforms generated from a fusion gene pair. An example of a fusion gene pair producing two isoforms is presented.

Among all the validated fusion gene partners, each gene was found to fuse with only one partner, except for LOC101495229, which formed fusions with both LOC101508351 and LOC101506473 (**Figure 2A**). The number of fusion isoforms generated by a fusion gene pair was inversely related to the number of such gene pairs detected, highlighting the specificity of junction sites within fusion genes. Among the validated fusions, all gene pairs had a single junction site, except the LOC101509445_LOC101509981 fusion, which exhibited two isoforms. In both isoforms, the breakpoint in the LOC101509445 gene was identical, whereas LOC101509981 contributed two distinct breakpoints, each located at the exon boundaries of two different alternatively spliced transcripts of the LOC101509981 gene. It’s a read-through fusion derived from two adjacent genes, where LOC101509445 is present on the reverse strand and LOC101509981 is present on the forward strand of chromosome 7. Here, the 5’ gene belongs to ABC transporter I family member 1, and the 3’ gene encodes for tRNA pseudouridine synthase. The two fusion events occur at the existing exon boundaries, hence producing in-frame fusions (**Figure 2B**).

To determine the fusion site within the parental genes, we analyzed the location of the breakpoint, whether it exists on the exon boundaries of both parental genes or one or none. It was observed that fusion transcripts where the breakpoint lies within the exon or in the UTR region were most abundant (57%), whereas fusion transcripts having a breakpoint at the exon border of either one or both parental genes were 25.3% and 17.6% respectively (**Supplementary Table S3**).

**Figure 3:**
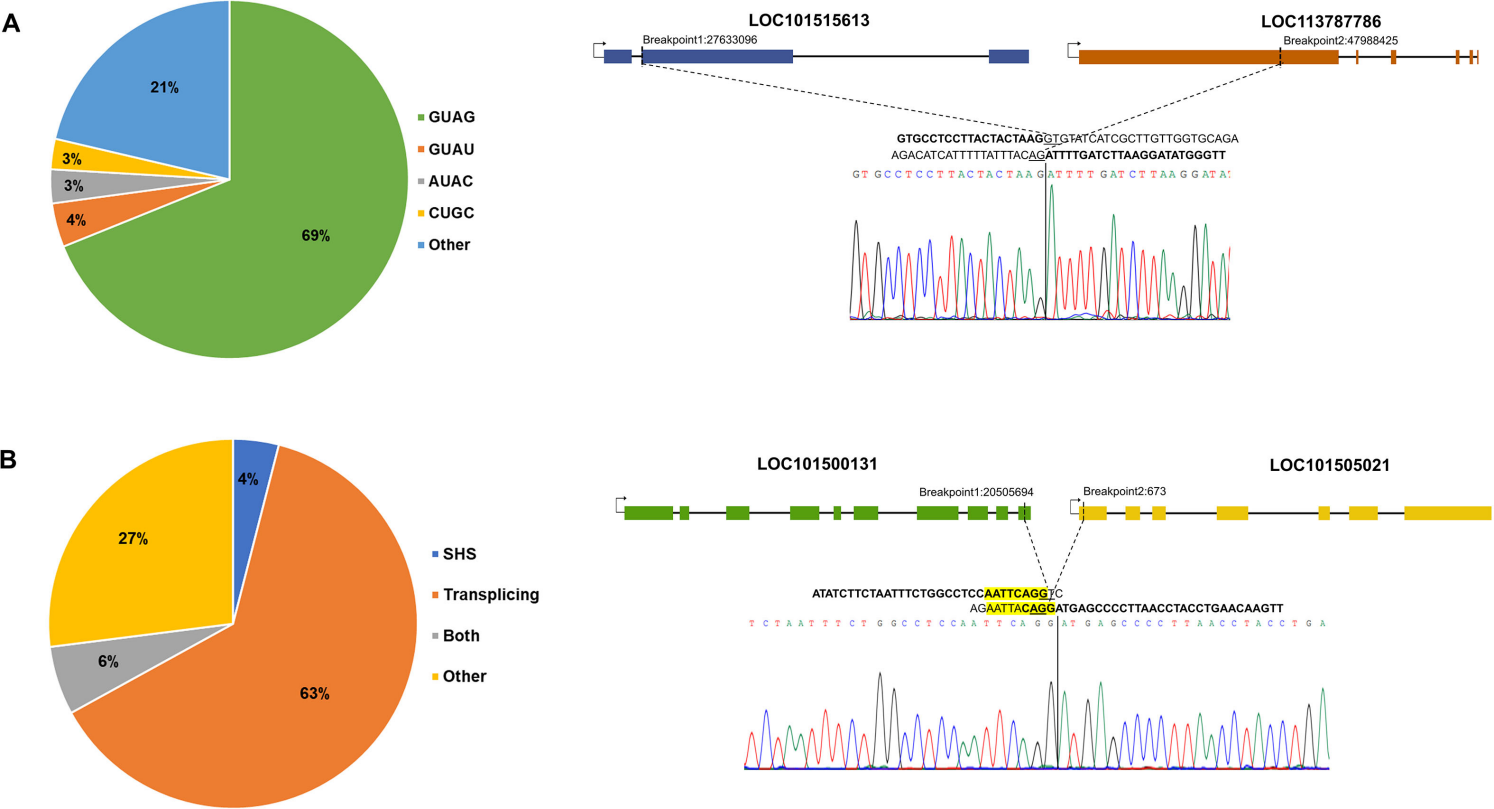
Junction site sequence analysis of fusion transcript, **(A)** The proportion of splice site patterns observed at fusion breakpoints is displayed, with an example fusion transcript demonstrating the canonical splice pattern (GT-AG) at the junction, **(B)** The contribution of different mechanisms to fusion generation is depicted, including trans-splicing and short homologous sequence (SHS) mediated fusion formation. An example fusion transcript displaying both canonical splice sites and SHS at the fusion junction is highlighted in yellow.

Trans-splicing is a known mechanism for fusion transcript formation (Li et al., 2009a). Junction pattern analysis showed that a significant portion of FTs exhibited canonical splice patterns GT-AG (69%); however, non-canonical splicing patterns were also found, such as GT-AT (4%), AT-AC (3%), CT-GC (3%), etc., implying apart from splicing, other unknown mechanisms may also contribute to fusion formation (**Figure 3A**). Another reported mechanism of fusion formation is transcriptional slippage mediated by the presence of short homologous sequences (SHSs) (Li et al., 2009b) at the junction, but less than 5% of total fusions exhibit SHSs at the breakpoints in *Arabidopsis*, soybean, rice, and maize (Cong et al., 2024). Similar results were observed in chickpea; 4% of total fusions showed SHSs at the junction, and 5.7% had both canonical splice sites and SHSs at their junction (**Supplementary Table S4**). All the experimentally validated fusions exhibited canonical splice sites at their junctions (e.g., LOC101515613_LOC113787786; **Figure 3A**), except for LOC101494819_LOC101493433, which showed a non-canonical GA-AG splice site. Additionally, three of the fusions also displayed short homologous sequences (SHSs) at the junctions, e.g., LOC101500131_LOC101505021 (**Figure 3B**).

Predicting the coding potential of transcripts is essential for understanding their functional roles. Fusion transcripts were thus classified as protein-coding or lncRNA based on the computational predictions. In our analysis, only 12.5% of the fusion transcripts were predicted to possess protein-coding ability, while the remaining 87.5% of the transcripts lacked coding potential and are unlikely to be translated into proteins (**Supplementary Table S5**). However, it is important to note that computational prediction of non-coding potential does not necessarily imply a lack of function. Recent studies have shown that some transcripts annotated as lncRNAs can be translated into biologically active micro-peptides (Ruiz-Orera and Albà, 2019; Choi et al., 2019; Li et al., 2022; Pan et al., 2022; Patraquim et al., 2022). These findings underscore that while the majority of fusion transcripts may not encode proteins, they could still play important regulatory or functional roles as lncRNAs.

### 3.3 Properties of genes involved in fusion formation

**Figure 4:**
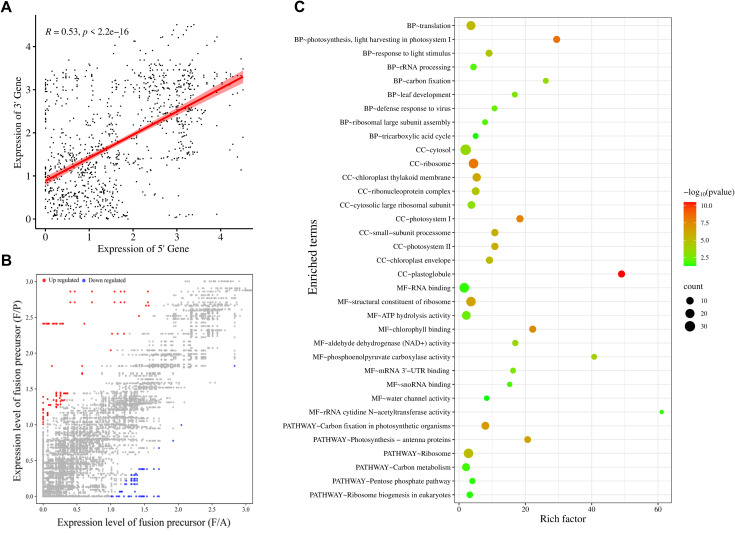
Functional categorization of parental genes involved in fusion, **(A)** Correlation between expression levels of 5’ and 3’ parental genes for each fusion event. Expression levels are presented as log (TPM + 1), with each point representing a fusion event. The R-value represents the Pearson correlation coefficient, **(B)** Scatterplot showing expression levels [log 2(TPM + 1)] of parental genes in samples with (F/P) and without the corresponding fusions (F/A). Colored dots indicate parental gene expression (fold change ≥ 2): grey for no change, blue for downregulation, and red for upregulation. **(C)** Gene Ontology analysis representing biological processes (BP), cellular component (CC), molecular function (MF), and KEGG pathway analysis of genes involved in fusion transcript formation. These plots were generated using SRplot (Tang et al., 2023).

Analysis of the transcriptome data showed a significant positive correlation (R = 0.53) between the expression of gene pairs involved in fusion formation, suggesting that these genes are linked in terms of expression (**Figure 4A**). Further to evaluate the impact of fusion formation on their parental genes, the expression levels of parental genes were compared between samples in which a particular fusion was present and those in which it was absent. In most samples, no significant change in the expression of fusion parental genes (~75%) was observed, suggesting that fusion formation may be independently regulated (**Figure 4B**). However, a subset of parental genes displayed differential expression in fusion-present samples, with 24 genes exhibiting ≥2-fold upregulation and only 3 showing ≥2-fold downregulation (**Supplementary Table S6**).

Fusion transcripts may retain or be associated with the biological functions of their parental genes. To gain insights into the potential functions of the fusion transcripts, we employed gene ontology (GO) and KEGG pathway analyses of parental genes involved in fusion formation. The most enriched biological GO terms were translation, photosynthesis, response to light stimulus, and rRNA processing (**Figure 4C**). The most enriched terms in the molecular process analysis showed that fusion parental genes are involved in the structural constituent of the ribosome and in binding, such as RNA binding and chlorophyll-binding, and in enzymatic activities such as ATP hydrolysis, carboxylase, and dehydrogenase. The most enriched cellular component GO terms were cytosol, ribosome, and chloroplast. Pathway enrichment analysis showed that these genes are related to the Ribosome, carbon metabolism, and photosynthesis. Conclusively, fusion transcripts originate from genes with diverse functions that are distributed across various cellular compartments, and also show important enzymatic and binding activities. Notably, photosynthesis and ribosome-associated genes are majorly enriched among fusion precursor genes (**Figure 4C**).

### 3.4 Validation of fusion transcripts from long-read RNA-Seq data

Transcript assembly of Illumina short reads to reconstruct full-length transcripts may introduce errors, particularly in regions with complex splicing patterns or novel splice sites. To overcome these challenges, long-read RNA sequencing technologies, such as PacBio, offer a significant advantage by capturing full-length transcripts in a single read, eliminating the need for assembly. For the identification of full-length fusion transcripts, publicly available long-read transcriptome sequencing datasets of *Cicer arietinum* were utilized. A BLASTn search of 200 bp fusion junction sequences, extracted from the results of fusion detection tools on Illumina datasets, against the PacBio long-read data identified a total of 95 fusion transcripts in the PacBio dataset, and their full length was predicted (**Supplementary Table S7**). The mean length of identified fusions was around 0.8 kb, and 70% of them had a length less than the mean length. We further compared the length of fusion transcripts and their parental genes and observed that the mean length of fusion transcripts was shorter than that of their parental genes (**Supplementary Figure S2**). This indicates that only specific portions of the parental genes are involved in fusion formation.

### 3.5 Intra- and interspecific conserved fusion events

**Figure 5:**
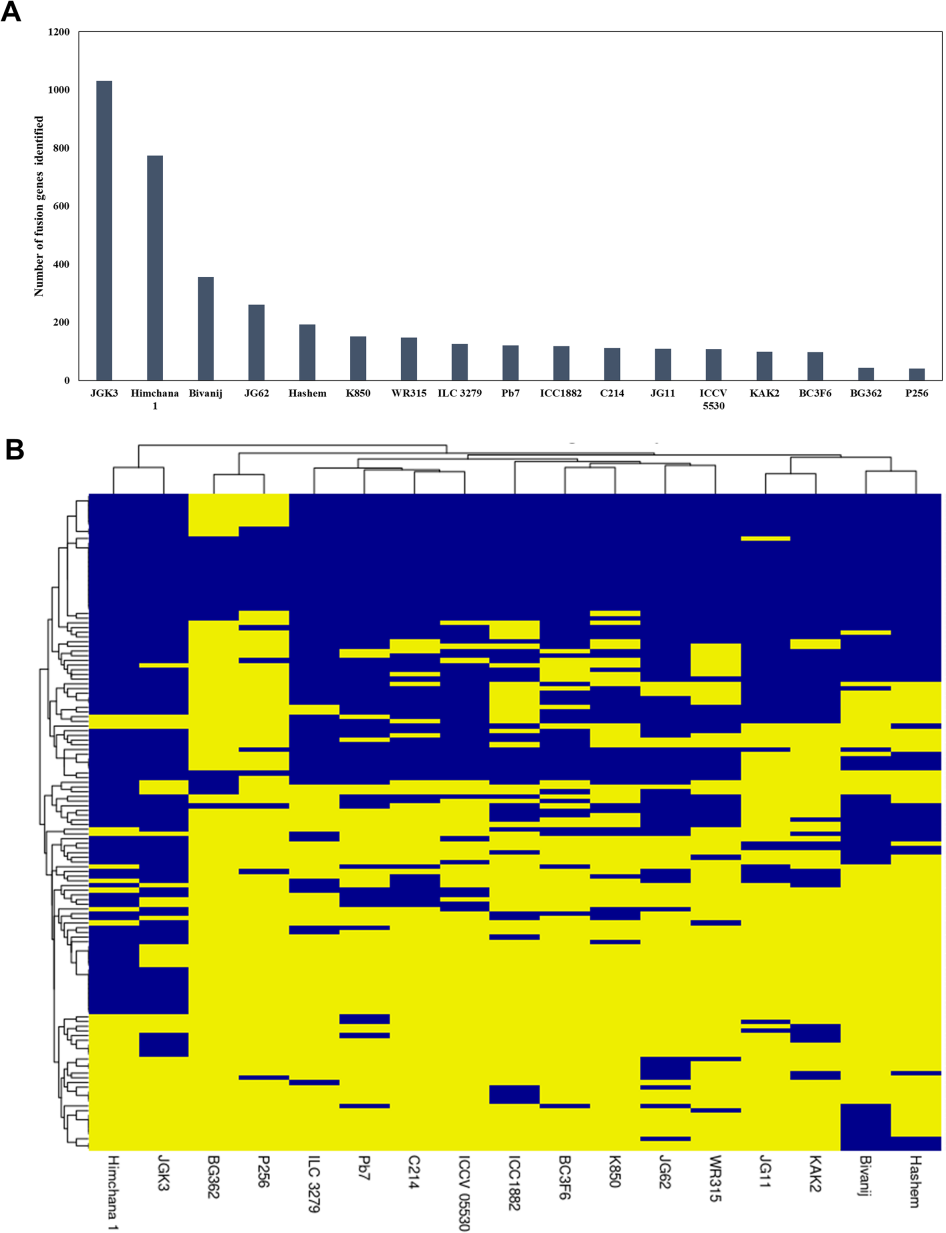
Fusion event profiles across different chickpea genotypes, **(A)** The number of fusion events identified in various chickpea genotypes, **(B)** Hierarchical clustering of 17 chickpea genotypes based on the presence or absence of fusion events identified in the ICC4958 genotype, with presence marked in blue and absence in yellow.

To explore intra-specific conserved fusion events, RNA-Seq data of 17 different chickpea genotypes were analyzed. The number of fusion transcripts identified across different genotypes varies significantly, ranging from 41 to 1031 (**Figure 5A**). This variation is likely attributed to differences in the number of samples analyzed for each genotype and the sequencing depth of the data. A fusion event was considered in a particular genotype if it was detected even in a single sample. After removing redundancy, interchromosomal fusion transcripts were found to be more prevalent than intrachromosomal fusion transcripts. Fusion genes identified in the ICC4958 genotype (269 fusion gene pairs) were searched across different genotypes (**Figure 5B**). Among these, 120 fusion gene pairs were conserved across multiple genotypes, while the rest were genotype-specific. Notably, 53 fusion gene pairs were detected in 10 or more genotypes, suggesting their potential biological significance and stability within the chickpea population.

To identify conserved FTs between Chickpea and *Arabidopsis*, FTs reported in AtFusionDB (Singh et al., 2019) were used. Among the 269 fusion genes identified in chickpea, 19 showed homology with 58 fusion events reported in *Arabidopsis* within AtFusionDB (**Supplementary Figure S3A**). Gene ontology analysis of these homologous genes revealed their involvement in essential biological processes, including metabolism and environmental responses (**Supplementary Figures S3B–D**). The presence of these fusion transcripts may play a critical role in enhancing biological functions linked to these processes.

### 3.6 Validation of fusion transcripts by qRT-PCR and Sanger sequencing

**Figure 6:**
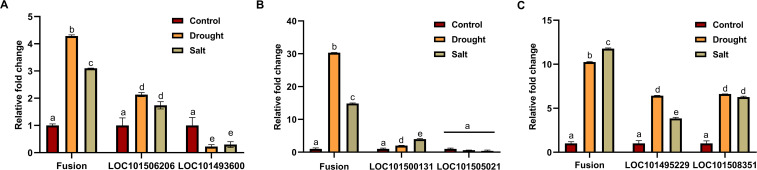
Relative expression of validated fusion transcripts and their precursors in *Cicer arietinum* under drought and salt stress conditions.

Expression of validated fusion transcripts under different abiotic stress and tissues by quantitative real-time PCR reveals that fusion transcripts are expressed at a very low level. Under different abiotic stress, three of the validated fusions (LOC101506206_LOC101493600, LOC101500131_LOC101505021, and LOC101495229_LOC101508351) showed differential expression, which implies that these fusions might play a crucial role during stress response (**Figure 6**). It was also observed that the relative fold change in expression of fusion transcripts was higher than that of their parental genes under stress conditions, suggesting that fusion transcript formation is not solely governed by the expression of precursor genes but may involve additional regulatory mechanisms (**Figure 6**). Fusion transcripts can originate from parental genes that exhibit diverse responses to stress, including downregulation, upregulation, or no significant change in expression. Expression analysis of fusion transcripts across different tissues revealed their tissue-specific nature. For example, the LOC101500131_LOC101505021 fusion transcript exhibited upregulation in bud and downregulation in stem and flower, relative to its expression in leaves (**Supplementary Figure S4**). The LOC101506206_LOC101493600 is an interchromosomal fusion derived from a gene encoding a folylpolyglutamate synthase-like enzyme located on chromosome 5 and an uncharacterized gene from chromosome 8. This fusion produces an in-frame transcript that arises from an existing splice site of the 5’ gene and a new splice site from the 3’ gene. Since folylpolyglutamate synthase is a single-subunit enzyme, this fusion might add new domains, which may enhance its enzymatic activity (Muralla et al., 2008; Li et al., 2016). Interestingly, under stress conditions, the fusion transcript exhibited a higher relative fold change in expression compared to either of its precursor genes: LOC101506206, which was upregulated, and LOC101493600, which was downregulated, compared to control conditions.

The LOC101500131_LOC101505021 fusion was identified across all RNA-Seq samples with varying levels of expression. This fusion is formed by the joining of exon 10 of the LOC101500131 gene to exon 1 of the LOC101505021 gene, both genes are present on the forward strand of DNA and are involved in similar functions that are anaphase-promoting complex. This fusion uses a canonical splice site, however, not at the existing exon boundaries of the parental genes, and generates a frameshift fusion. Interestingly, this fusion showed significant upregulation in both drought and salt stresses as compared to the control condition. In contrast, the expression of the fusion precursor genes remains relatively unchanged in response to stress. Therefore, the regulation of the LOC101500131_LOC101505021 fusion appears to be independent of its parental genes.

The LOC101495229_LOC101508351 fusion is formed by joining exon 2 from LOC101495229 and exon 1 from LOC101508351. One of the parental genes acts as a ubiquitin ligase, while the other remains uncharacterized. This fusion occurs at the exon boundary of the LOC101508351 gene and uses a new canonical splice site from the LOC101495229 gene. Both fusion transcript and its parental genes are stress-responsive; however, the relative change in fusion transcript expression under stress conditions is higher compared to parental genes.

### 3.7 Comprehensive assessment of fusion detection tools for *Cicer arietinum*

While many tools are available for fusion detection in humans, none of them is exclusively designed for plants, except EricScript-Plants (Benelli et al., 2012), which is limited to plant genomes available at Ensembl, and hence cannot be used for chickpea. In this study, we initially employed FusionMap, STAR-Fusion, and MapSplice for the comprehensive detection of fusion transcripts in chickpea. Further, a comparative analysis of five fusion detection algorithms was conducted to identify the best-performing tool for fusion detection for this plant species. This revealed the following order of tools based on sensitivity and F-measure: FusionMap > STAR-Fusion > MapSplice > SQUID > Tophat-Fusion. A similar ranking was observed with public data, except that STAR-Fusion performed better than FusionMap with public data (**Supplementary Figures S5A, B**). Of note, there is a small overlap in the fusions detected by these tools in both our dataset and public data (**Supplementary Figure S5C, D**). This could be due to false discoveries associated with individual software packages, or the fact that none of the tools is inclusive. Fusion gene pairs LOC101506206_LOC101493600 and LOC101503481_LOC101494793 were commonly detected by 3 out of the 5 fusion detection tools.

This analysis suggests that FusionMap detected the maximum number of true fusions, but it also identified a large number of false fusions, whereas STAR-Fusion identified fewer false fusions compared to FusionMap. Hence, FusionMap is the best performer in terms of sensitivity, whereas STAR-Fusion is the best performer in terms of precision. Hence, a combination of STAR-Fusion and FusionMap is a better choice for fusion transcript detection from RNA-Seq data for *Cicer* until more efficient tools become available.

Another factor that affects the detection of FTs from RNA-Seq data is the sequencing depth. It was observed that samples with higher sequencing depth showed a larger number of fusions than those with lower sequencing depth. For instance, in the P3 sample, the number of FTs detected is more than that in CS2 because the sequencing depth of P3 is higher than CS2 (**Supplementary Table S2**).

## 4 Discussion

In recent years, it’s already established that the fusion transcripts are not exclusive to tumors but also occur in normal human tissues and a wide variety of species (Babiceanu et al., 2016; Cong et al., 2024). Although significant progress has been made in mammalian fusion transcript research, studies on fusion transcripts in plants remain limited. While some studies have been conducted on model species such as *Arabidopsis* and rice, a comprehensive investigation of plant fusion transcripts is still lacking. A few plant-specific fusion transcripts databases are also available (Singh et al., 2019; Arya et al., 2024). Recently, a comprehensive profiling of fusion transcripts was conducted using public RNA-Seq datasets of Arabidopsis, Rice, and Chickpea (Chitkara et al., 2024). Most fusion transcripts were detected in only a single sample, raising concerns about their biological reproducibility and potential as artifacts. The current study addresses this gap through the use of in-house generated RNA-Seq datasets under defined abiotic stress conditions and across tissue types.

Here, we present the genome-wide identification of fusion transcripts in *Cicer arietinum* and explore their potential functional roles. Our study enhances the current understanding of fusion transcripts in legume plants. Notably, we found that interchromosomal fusion transcripts are more prevalent than those of intrachromosomal fusions in Chickpea, a pattern consistent with maize and soybean. In contrast, rice and *Arabidopsis* exhibit a higher frequency of intrachromosomal fusions (Cong et al., 2024). This indicates that the proportion of interchromosomal and intrachromosomal fusion transcripts is variable among different plants and may be influenced by several factors, including the gene density and compactness of the genome. The majority of gene pairs involved in fusion formation have no other partner genes, with only a few showing fusion isoforms, highlighting the specificity of these events. Additionally, we identified fusion transcripts that are tissue-specific or induced under specific stress conditions. Expression correlation analysis of genes involved in fusions revealed a correlation coefficient (r) of 0.5, aligning with the recent report that suggests genes with similar transcriptional activity are more likely to participate in conserved fusion events (Cong et al., 2024). Most parental genes involved in these fusions are multi-exonic and protein-coding, but only 12.5% of fusion transcripts showed protein-coding ability, while 87.5% were non-coding. Despite lacking coding capacity, these may still have regulatory or functional roles as lncRNAs.

Fusion transcript identification in different genotypes of chickpea revealed that some fusions are conserved across all genotypes, while others are specific to individual genotypes, indicating a link between genomic variation and fusion transcript occurrence. Homologous fusion events between *Cicer arietinum* and *Arabidopsis* suggest that the parental genes involved play key roles in essential biological processes, underscoring the conservation of fusion events across different plant species.

Most fusion breakpoints exhibited canonical splice sites at the fusion junctions, implying their generation via the splicing mechanism. However, a few fusions showed overlapping sequences at the breakpoints, suggesting that SHSs also contribute to the formation of these fusions in *Cicer arietinum*. Further investigation revealed that SHSs differ among fusion transcripts and are mostly<10 bp in length. Due to the short reads generated by Illumina sequencing, full-length fusion transcripts could not be reliably detected. To address this limitation, we employed long-read RNA sequencing to identify the full-length structure of fusion transcripts. Of the 328 fusion events initially identified, 95 were confirmed in the long-read RNA-Seq data, likely due to the lower sequencing depth of this dataset.

Out of the predicted fusion transcripts, we confirmed the existence of 10 FTs, marking the first experimental validation of fusion transcripts in *Cicer arietinum*. Notably, three of these fusions were stress-responsive and exhibited upregulation in drought and salt stress, suggesting a potential function in the plant’s stress response mechanisms. However, despite thorough validation efforts, we were unable to identify any fusion transcripts that were exclusively expressed under a specific stress and absent in the control condition. This indicates that while fusion transcripts might be involved in the stress response, their expression may not be strictly limited to stress conditions. These fusions may represent a broader regulatory mechanism that operates under both normal and stress conditions but becomes more pronounced in response to stress. The confirmation of stress-responsive fusion transcripts paves the way for exploring the functional implications of these fusions in stress adaptation and resilience. Future research could focus on dissecting the biological roles of these fusions, including their impact on gene expression and protein function under stress conditions. Additionally, investigating the potential regulatory networks and pathways associated with these fusions could provide a profound understanding of how *Cicer arietinum* adapts to environmental challenges.

## 5 Conclusion

This study systematically reveals the transcriptomic complexity arising due to fusion events in chickpea by combining transcriptomic datasets and experimental validation across diverse tissues (leaf, stem, bud, flower, and pod) and conditions (drought and salinity). Transcriptome analysis revealed the involvement of both coding and non-coding genes in the fusion events located either in close proximity or distant from each other. The parental genes involved in these fusions are associated with diverse biological pathways, suggesting the potential diverse roles of the fusion events that need to be further experimentally evaluated. Comparative analysis revealed both inter- and intraspecific conservation of fusion events, while also uncovering intra-species diversity, thereby underscoring their evolutionary significance. Expression analysis revealed stress-responsive fusion transcripts, indicating their potential regulatory roles under abiotic stress. Overall, this work provides a comprehensive report of fusion transcript diversity in chickpea and offers a valuable foundation for future functional studies aimed at elucidating their biological and adaptive relevance.

## Data Availability

The datasets presented in this study are publicly available. This data can be found here: https://www.ncbi.nlm.nih.gov/sra, accession numbers PRJNA613159, PRJNA288321 and PRJNA1296865.

## Supplementary material

materialThe Supplementary Material for this article can be found online at: https://www.frontiersin.org/articles/10.3389/fpls.2025.1677098/full#supplementary-material

Supplementary Figure 1 Chromatogram of 10 validated fusion transcripts from chickpea **(A–J)**, where black line marks the junction site between the two fusion genes.

Supplementary Figure 2 Box plot depicting the relation between the predicted length of fusion transcripts and their parental genes.

Supplementary Figure 3 Inter-specific conserved fusion events, **(A)** Venn diagram showing orthologous fusion events shared between *Cicer arietinum* and *Arabidopsis thaliana*. **(B–D)** Gene Ontology (GO) enrichment analysis of orthologous fusion gene pairs, highlighting enriched categories in, **(B)** Molecular Function, **(C)** Biological Process, and **(D)** Cellular Component.

Supplementary Figure 4 Relative expression of three validated fusion transcripts **(A–C)** in *Cicer arietinum* across different tissues.

Supplementary Figure 5 Benchmark analysis of fusion detection tools using in-house generated RNA-seq data and publicly available RNA-seq data from chickpea, **(A)** Sensitivity of fusion detection tools evaluated for both datasets, calculated as Sensitivity (%) = (TP/TF) * 100, where TP represents true positives and TF is the total number of fusions, **(B)** F-measure for each tool, calculated as F-measure = 2 * (Sensitivity * Precision)/(Sensitivity/Precision), where Precision (%) = TP/(TP + FP) * 100. **(C, D)** Venn diagrams showing the overlapping fusion transcripts identified by different fusion detection tools in the in-house generated RNA-seq data **(C)** and the publicly available RNA-seq data, **(D),** highlighting common fusions across the tools. The performance metrics (sensitivity, specificity, and F-measure) were calculated based on the identification of validated fusion transcripts (true fusions) across all tools.

Supplementary Figure 6 Melt curve plot for the endogenous control gene and stress-responsive fusion transcripts, along with the primer amplification efficiency (%).

Supplementary Table 1 Summary of RNA-Seq reads and mapping statistics.

Supplementary Table 2 Summary of the number of fusion transcripts detected in each sample by three fusion detection methods and the sequencing depth of each sample.

Supplementary Table 3 List of fusion transcripts detected in RNA-Seq samples.

Supplementary Table 4 Information regarding Short homologous sequences (SHSs) detected at the junction of the fusion transcript.

Supplementary Table 5 Predicted coding potential of fusion transcripts.

Supplementary Table 6 List of differentially expressed genes in samples with and without fusion transcripts.

Supplementary Table 7 List of fusion transcripts detected in Long-read RNA-Seq data.

Supplementary Table 8 List of RNA-Seq samples used to study intra-specific diversity of fusion transcripts.

Supplementary Table 9 List of primers used for qRT-PCR of fusion transcripts and their parental genes.
