# Supplementary figures and images for: Molecular and expression analyses indicate the role of fusion transcripts in mediating abiotic stress responses in chickpea
Source: Front Plant Sci. 2025 Oct 31;16:1677098. doi: 10.3389/fpls.2025.1677098 (PMC12615446; doi:10.3389/fpls.2025.1677098)

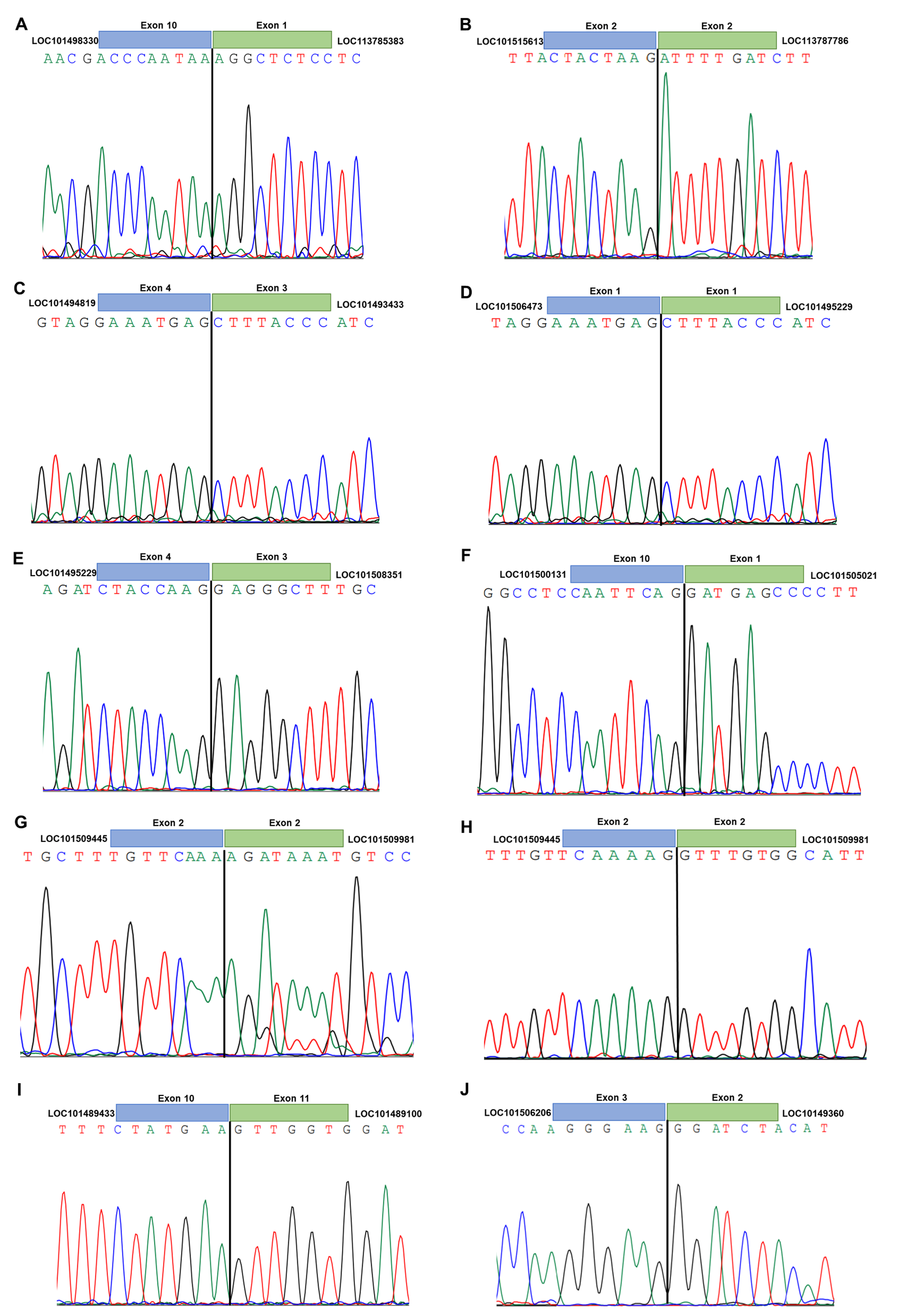

Supplement: Supplementary Figure 1 — Chromatogram of 10 validated fusion transcripts from chickpea (A–J), where black line marks the junction site between the two fusion genes. [file Image1.tif]

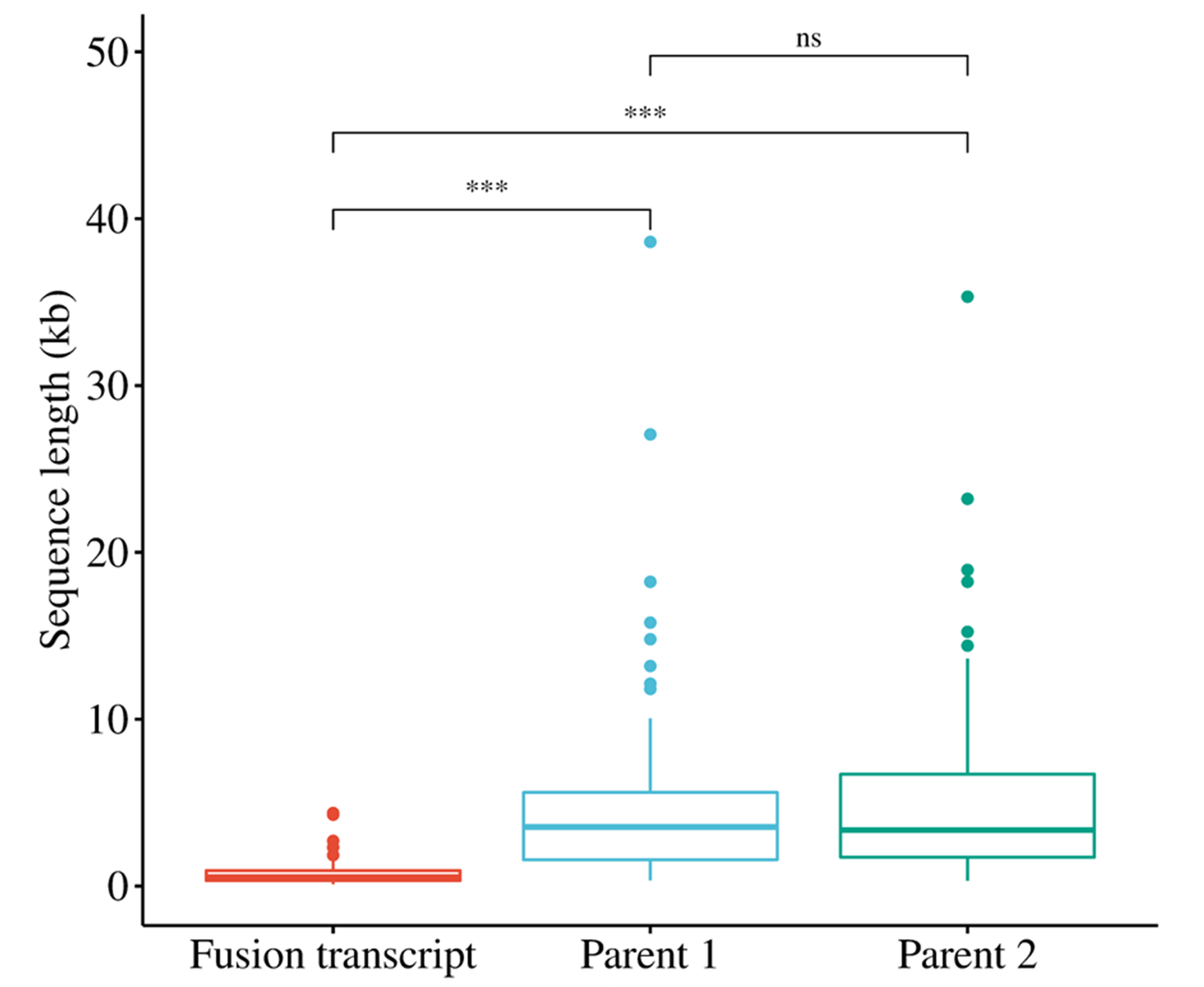

Supplement: Supplementary Figure 2 — Box plot depicting the relation between the predicted length of fusion transcripts and their parental genes. [file Image2.tif]

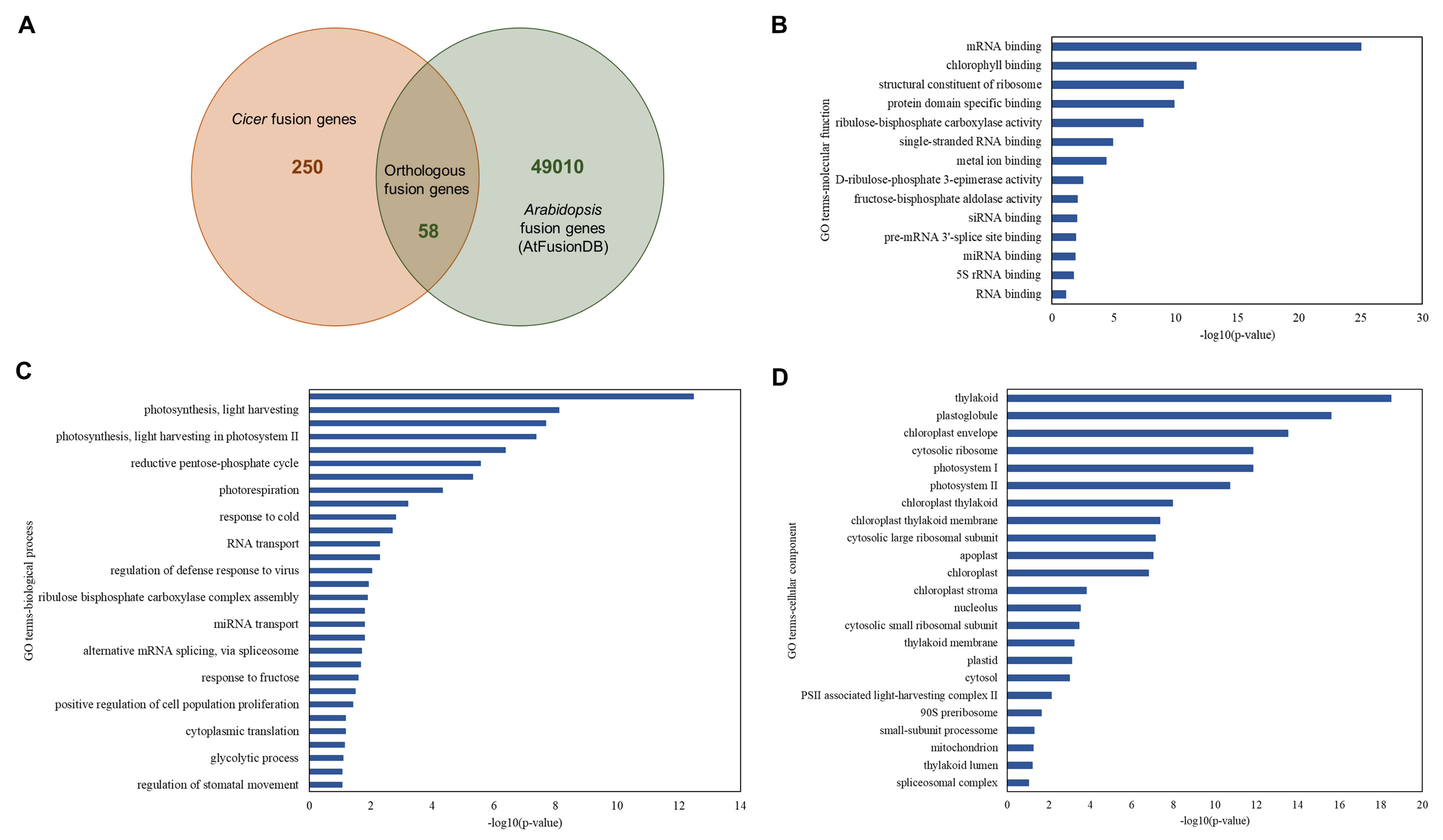

Supplement: Supplementary Figure 3 — Inter-specific conserved fusion events, (A) Venn diagram showing orthologous fusion events shared between Cicer arietinum and Arabidopsis thaliana. (B–D) Gene Ontology (GO) enrichment analysis of orthologous fusion gene pairs, highlighting enriched categories in, (B) Molecular Function, (C) Biological Process, and (D) Cellular Component. [file Image3.tif]

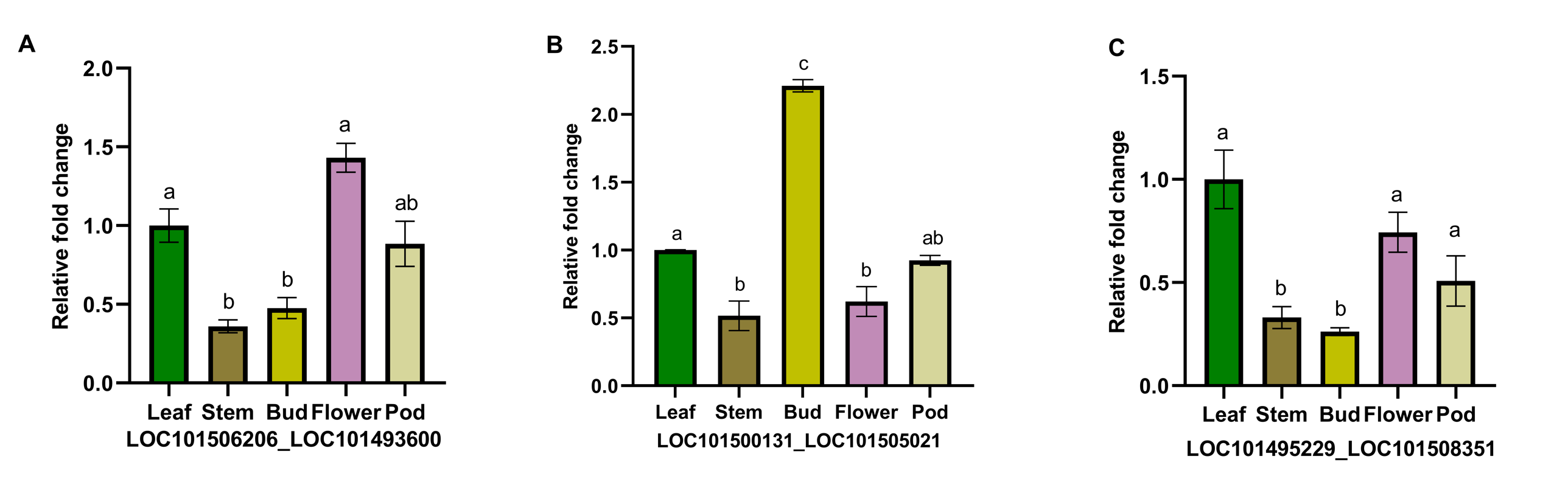

Supplement: Supplementary Figure 4 — Relative expression of three validated fusion transcripts (A–C) in Cicer arietinum across different tissues. [file Image4.tif]

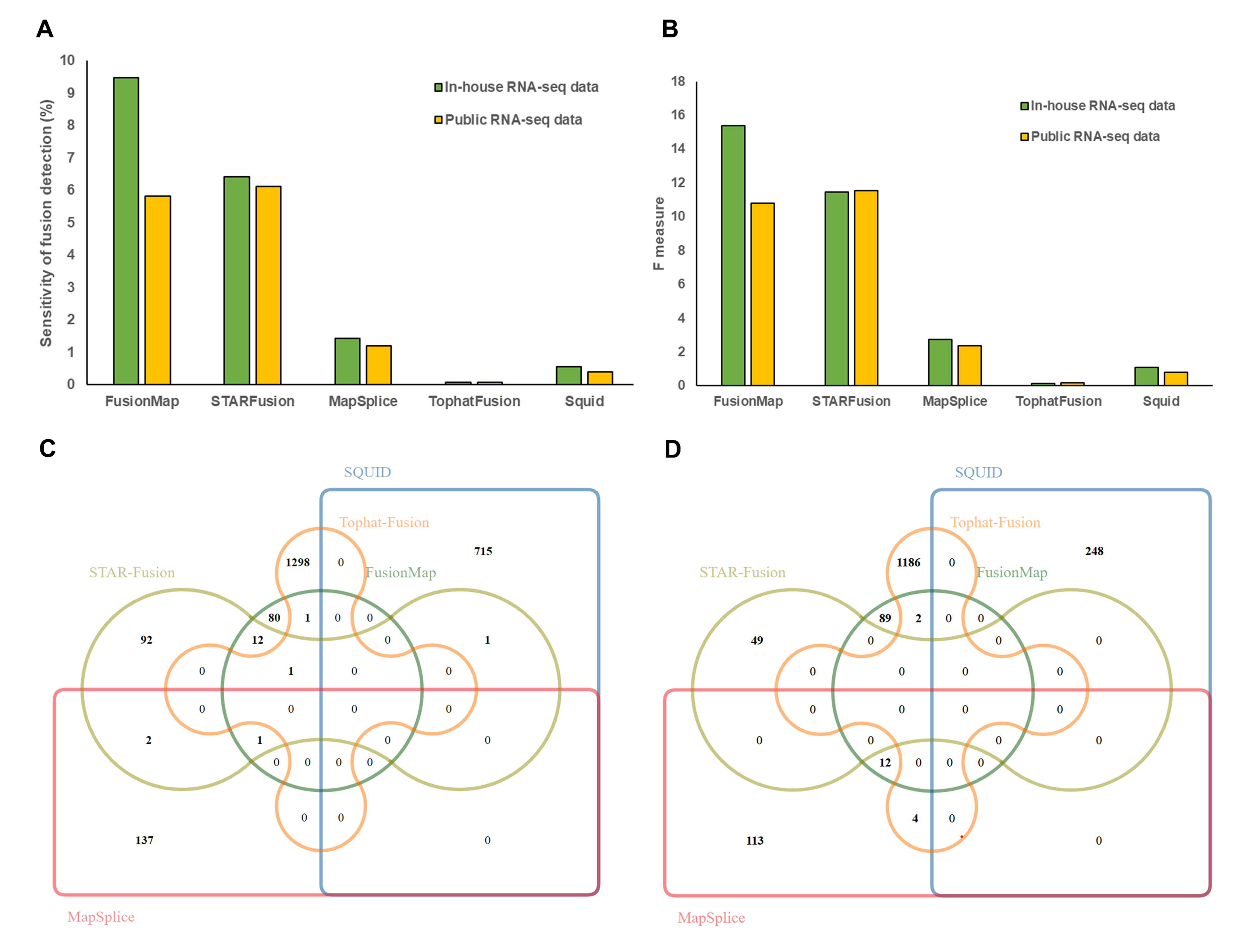

Supplement: Supplementary Figure 5 — Benchmark analysis of fusion detection tools using in-house generated RNA-seq data and publicly available RNA-seq data from chickpea, (A) Sensitivity of fusion detection tools evaluated for both datasets, calculated as Sensitivity (%) = (TP/TF) * 100, where TP represents true positives and TF is the total number of fusions, (B) F-measure for each tool, calculated as F-measure = 2 * (Sensitivity * Precision)/(Sensitivity/Precision), where Precision (%) = TP/(TP + FP) * 100. (C, D) Venn diagrams showing the overlapping fusion transcripts identified by different fusion detection tools in the in-house generated RNA-seq data (C) and the publicly available RNA-seq data, (D), highlighting common fusions across the tools. The performance metrics (sensitivity, specificity, and F-measure) were calculated based on the identification of validated fusion transcripts (true fusions) across all tools. [file Image5.tif]
