# Supplementary material for: Molecular and expression analyses indicate the role of fusion transcripts in mediating abiotic stress responses in chickpea
Source: Front Plant Sci. 2025 Oct 31;16:1677098. doi: 10.3389/fpls.2025.1677098 (PMC12615446; doi:10.3389/fpls.2025.1677098)
Supplement: Supplementary Table 1 — Summary of RNA-Seq reads and mapping statistics. [file Table1.docx]

**Table S1.** Summary of RNA-Seq reads and mapping statistics.

| **Sample ID** | **Description** | **Total reads before filtering** | **Total bases before filtering** | **Total reads after filtering** | **Total bases after filtering** | **Overall mapping rate** |
| --- | --- | --- | --- | --- | --- | --- |
| B1 | bud1 | 32.097466 M | 4.814620 G | 31.798002 M | 4.665035 G | 97.38% |
| B2 | bud2 | 39.589018 M | 5.938353 G | 39.274374 M | 5.783985 G | 97.75% |
| B3 | bud3 | 32.111594 M | 4.848851 G | 31.439312 M | 4.405996 G | 96.60% |
| CD1 | control drought1 | 35.675820 M | 5.387049 G | 35.379608 M | 5.106564 G | 96.89% |
| CD2 | control drought2 | 35.155350 M | 5.308458 G | 34.895670 M | 4.974223 G | 97.01% |
| CD3 | control drought3 | 35.272586 M | 5.290888 G | 35.040190 M | 5.186991 G | 95.31% |
| CS1 | control salt1 | 28.409916 M | 4.289897 G | 28.088466 M | 3.974790 G | 96.99% |
| CS2 | control salt2 | 23.759012 M | 3.587611 G | 23.537146 M | 3.350033 G | 96.77% |
| CS3 | control salt3 | 34.412248 M | 5.161837 G | 34.053352 M | 5.022196 G | 97.47% |
| D1 | drought1 | 29.990286 M | 4.498543 G | 29.663260 M | 4.377915 G | 97.52% |
| D2 | drought2 | 30.762700 M | 4.614405 G | 30.452014 M | 4.511143 G | 97.29% |
| D3 | drought3 | 32.090942 M | 4.813641 G | 31.633712 M | 4.665287 G | 97.05% |
| F1 | flower1 | 28.445162 M | 4.266774 G | 28.235434 M | 4.132264 G | 97.36% |
| F2 | flower2 | 35.872482 M | 5.380872 G | 35.545986 M | 5.204315 G | 95.22% |
| F3 | flower3 | 31.034542 M | 4.686216 G | 30.524134 M | 4.104437 G | 96.30% |
| L1 | leaf1 | 27.014486 M | 4.052173 G | 26.794922 M | 3.946489 G | 97.63% |
| L2 | leaf2 | 32.510794 M | 4.876619 G | 32.231424 M | 4.758872 G | 97.40% |
| P1 | pod1 | 34.432722 M | 5.199341 G | 34.013796 M | 4.835398 G | 96.94% |
| P2 | pod2 | 40.797534 M | 6.160428 G | 40.427928 M | 5.746738 G | 96.20% |
| P3 | pod3 | 38.289934 M | 5.781780 G | 37.990414 M | 5.443072 G | 97.04% |
| S1 | salt1 | 25.885696 M | 3.882854 G | 25.594944 M | 3.796070 G | 97.47% |
| S2 | salt2 | 37.785730 M | 5.705645 G | 37.232842 M | 5.148091 G | 96.98% |
| S3 | salt3 | 24.385190 M | 3.682164 G | 24.004768 M | 3.304218 G | 97.14% |
| ST1 | stem1 | 41.337854 M | 6.242016 G | 40.896440 M | 5.510618 G | 96.93% |
| ST2 | stem2 | 39.581018 M | 5.976734 G | 38.907446 M | 5.553084 G | 96.96% |
| ST3 | stem3 | 35.630652 M | 5.380228 G | 35.153606 M | 4.967206 G | 97.07% |
