# Supplementary material for: Molecular and expression analyses indicate the role of fusion transcripts in mediating abiotic stress responses in chickpea
Source: Front Plant Sci. 2025 Oct 31;16:1677098. doi: 10.3389/fpls.2025.1677098 (PMC12615446; doi:10.3389/fpls.2025.1677098)
Supplement: Supplementary Table 2 — Summary of the number of fusion transcripts detected in each sample by three fusion detection methods and the sequencing depth of each sample. [file Table2.docx]

**Table S2.** Summary of the number of fusion transcripts detected in each sample by three fusion detection methods and sequencing depth of each sample.

| **Sample** | **Description** | **Sequencing depth** | **FusionMap** | **STAR-Fusion** | **MapSplice** |
| --- | --- | --- | --- | --- | --- |
| B1 | bud1 | 8.788686888 | 21 | 33 | 19 |
| B2 | bud2 | 10.89673135 | 22 | 33 | 19 |
| B3 | bud3 | 8.300670686 | 19 | 29 | 14 |
| CD1 | control drought1 | 9.620504898 | 23 | 30 | 35 |
| CD2 | control drought2 | 9.371181236 | 24 | 29 | 20 |
| CD3 | control drought3 | 9.772025245 | 27 | 33 | 19 |
| CS1 | control salt1 | 7.488300678 | 14 | 26 | 16 |
| CS2 | control salt2 | 6.311290505 | 18 | 22 | 15 |
| CS3 | control salt3 | 9.46155991 | 20 | 25 | 18 |
| D1 | drought1 | 8.247767521 | 17 | 21 | 14 |
| D2 | drought2 | 8.498762246 | 17 | 23 | 12 |
| D3 | drought3 | 8.789161643 | 14 | 25 | 17 |
| F1 | flower1 | 7.784973625 | 18 | 30 | 18 |
| F2 | flower2 | 9.804662773 | 26 | 26 | 18 |
| F3 | flower3 | 7.732548983 | 17 | 23 | 11 |
| L1 | leaf1 | 7.434983044 | 20 | 28 | 22 |
| L2 | leaf2 | 8.965470987 | 26 | 24 | 26 |
| P1 | pod1 | 9.10964205 | 16 | 31 | 30 |
| P2 | pod2 | 10.82655991 | 24 | 28 | 30 |
| P3 | pod3 | 10.25446873 | 25 | 42 | 31 |
| S1 | salt1 | 7.151601356 | 15 | 20 | 17 |
| S2 | salt2 | 9.698739638 | 19 | 26 | 20 |
| S3 | salt3 | 6.224977393 | 17 | 22 | 14 |
| ST1 | stem1 | 10.38172193 | 21 | 32 | 21 |
| ST2 | stem2 | 10.4617257 | 19 | 33 | 35 |
| ST3 | stem3 | 9.357961567 | 20 | 27 | 22 |
| Total fusion detected |  |  | 496 | 721 | 533 |
| No. of unique FTs |  |  | 95 | 109 | 140 |
