# Supplementary material for: Molecular and expression analyses indicate the role of fusion transcripts in mediating abiotic stress responses in chickpea
Source: Front Plant Sci. 2025 Oct 31;16:1677098. doi: 10.3389/fpls.2025.1677098 (PMC12615446; doi:10.3389/fpls.2025.1677098)
Supplement: Supplementary Table 3 — List of fusion transcripts detected in RNA-Seq samples. [file Table3.docx]

**Table S3.** List of fusion transcripts detected in RNA-Seq samples.

| **Chromosome 1** | **Breakpoint 1** | **Strand 1** | **Chromosome 2** | **Breakpoint 2** | **Strand 2** | **Gene 1** | **Gene 2** | **Splice site location** |
| --- | --- | --- | --- | --- | --- | --- | --- | --- |
| 2 | 2062657 | - | 3 | 36308938 | + | LOC101494223 | LOC101504783 | None |
| 2 | 23931340 | + | NW_004517187.1 | 5631 | + | LOC101512687 | LOC101503191 | Both |
| 2 | 23933574 | + | NW_004517187.1 | 5631 | + | LOC101512687 | LOC101503191 | Both |
| 2 | 23933994 | + | NW_004517187.1 | 5631 | + | LOC101512687 | LOC101503191 | None_Exon |
| 2 | 247417 | + | 2 | 29700242 | + | LOC101499528 | LOC101494120 | None |
| 2 | 247417 | + | 6 | 1116301 | - | LOC101499528 | LOC101512000 | None |
| 2 | 247461 | - | 2 | 257539 | + | LOC101499528 | LOC105851539 | None |
| 2 | 25534571 | - | 7 | 22344920 | - | LOC113785449 | LOC101494531 | None |
| 2 | 257048 | + | 6 | 1116319 | - | LOC105851539 | LOC101512000 | None |
| 2 | 257066 | + | 2 | 29700242 | + | LOC105851539 | LOC101494120 | None |
| 2 | 257066 | + | 6 | 1116301 | - | LOC105851539 | LOC101512000 | None |
| 2 | 30028516 | + | 4 | 11451527 | + | LOC101504009 | LOC101493413 | None |
| 2 | 30144899 | - | 4 | 2023971 | + | LOC101511831 | LOC101502954 | None |
| 2 | 30744589 | - | 7 | 13555156 | - | LOC101504980 | LOC101509554 | None |
| 2 | 30965639 | - | 3 | 34452352 | - | LOC101514211 | LOC101508956 | None |
| 2 | 31356943 | + | 3 | 39264029 | - | LOC101493912 | LOC101496628 | None_Exon |
| 2 | 31358189 | + | 3 | 21635029 | - | LOC101493912 | LOC101509484 | None_Exon |
| 2 | 31358189 | + | 3 | 21635586 | - | LOC101493912 | LOC101509484 | None_Exon |
| 2 | 3343313 | - | 6 | 14022203 | + | LOC101514962 | LOC101496011 | None |
| 2 | 3343375 | + | 6 | 14022305 | - | LOC101514962 | LOC101496011 | None |
| 2 | 3343694 | - | 6 | 14021908 | + | LOC101514962 | LOC101496011 | None |
| 2 | 3343711 | + | 6 | 14021889 | - | LOC101514962 | LOC101496011 | None |
| 1 | 13497308 | + | 2 | 30479154 | + | LOC101510411 | LOC101495619 | None |
| 2 | 34908711 | + | 2 | 34943519 | + | LOC113785512 | LOC101497027 | Both |
| 2 | 35441273 | + | 4 | 40170427 | + | LOC101506059 | LOC101500851 | Both |
| 2 | 36051172 | + | 2 | 3642870 | - | LOC101505628 | LOC101495178 | None |
| 2 | 6229425 | - | 1 | 32602422 | - | LOC101498330 | LOC113785383 | None |
| 2 | 6381127 | - | 5 | 45803311 | + | LOC101499938 | LOC101503085 | None |
| 2 | 6381228 | + | 2 | 36191405 | - | LOC101499938 | LOC101514326 | None |
| 2 | 6414031 | + | 7 | 13670917 | + | LOC101501330 | LOC101512251 | None |
| 2 | 7569350 | + | 7 | 18818298 | - | LOC101501648 | LOC101499935 | Exon_None |
| 2 | 7668307 | - | 6 | 1932646 | - | LOC101502810 | LOC101511778 | None |
| 2 | 7668365 | - | 4 | 14069897 | - | LOC101502810 | LOC101507074 | None |
| 2 | 7668383 | - | 4 | 11451540 | + | LOC101502810 | LOC101493413 | None |
| 2 | 7668387 | - | 6 | 19726142 | - | LOC101502810 | LOC101494085 | None |
| 2 | 7668504 | - | 1 | 6097477 | + | LOC101502810 | LOC101494711 | None |
| 2 | 7668518 | - | 8 | 13248333 | - | LOC101502810 | LOC101495739 | None |
| 2 | 8273513 | - | 6 | 9515613 | + | LOC101494112 | UBQ5 | None |
| 2 | 8294351 | - | 6 | 23393185 | + | LOC101494736 | LOC101489111 | None |
| 2 | 8295603 | - | 6 | 23393179 | + | LOC101494736 | LOC101489111 | Exon_None |
| 3 | 16533516 | + | 1 | 2538836 | + | LOC101501345 | LOC101500252 | None_Exon |
| 3 | 23900664 | - | 2 | 8516622 | - | LOC101506274 | LOC101501235 | None_Exon |
| 3 | 26932908 | - | 5 | 33690951 | + | LOC101491644 | LOC101505889 | None |
| 3 | 27404363 | + | 8 | 5439100 | + | LOC101508430 | LOC101515298 | None |
| 3 | 27784879 | - | 3 | 34452357 | - | LOC101497592 | LOC101508956 | None |
| 3 | 27845438 | - | 2 | 27608496 | + | LOC101498915 | LOC101509145 | Exon_None |
| 3 | 27956976 | + | 3 | 31303423 | + | LOC101501673 | LOC101515202 | None |
| 3 | 28523480 | + | 7 | 40734451 | - | LOC101501261 | LOC101510205 | None |
| 3 | 2879883 | - | 1 | 6097564 | + | LOC101491737 | LOC101494711 | None |
| 1 | 16466208 | + | 6 | 14021894 | - | LOC101499819 | LOC101496011 | None |
| 3 | 28995363 | - | 3 | 29007310 | - | LOC101489274 | LOC101489596 | None |
| 3 | 29226306 | + | 2 | 24651696 | + | LOC105851812 | LOC113785432 | Exon_None |
| 1 | 16466254 | + | 6 | 14021848 | - | LOC101499819 | LOC101496011 | None |
| 3 | 31623291 | + | 2 | 31359283 | + | LOC101501466 | LOC101493912 | Exon_None |
| 3 | 32961388 | - | 6 | 19735634 | + | LOC101493176 | LOC101504207 | None |
| 3 | 34502846 | + | NW_004522750.1 | 17073 | - | LOC101509269 | LOC101496647 | None |
| 3 | 35829859 | + | 4 | 40170427 | + | LOC101507895 | LOC101500851 | Both |
| 3 | 36712301 | + | 4 | 43532656 | + | LOC101497170 | LOC113786100 | Both |
| 3 | 36712301 | + | 6 | 32237813 | + | LOC101497170 | LOC101514815 | Exon_None |
| 3 | 37683093 | + | NW_004516029.1 | 234017 | - | LOC113785842 | LOC101490259 | Both |
| 3 | 39340361 | + | 3 | 39300069 | + | LOC101501581 | LOC101498254 | Both |
| 3 | 39751662 | - | 5 | 30675509 | - | LOC101499472 | LOC101512214 | None |
| 3 | 39988535 | + | 1 | 958 | - | LOC101506836 | LOC101497325 | None |
| 3 | 6965732 | + | 3 | 6838668 | - | LOC101501663 | LOC101500070 | None |
| 3 | 974327 | + | 1 | 3266649 | + | LOC113785863 | LOC101504403 | None |
| 4 | 11378175 | + | 4 | 45752819 | + | LOC101488621 | LOC101509617 | Both |
| 4 | 11724007 | + | 6 | 58255084 | + | LOC101500209 | LOC101506557 | None |
| 4 | 11724046 | + | 3 | 26933074 | - | LOC101500209 | LOC101491644 | None |
| 4 | 11724046 | + | 7 | 1701609 | - | LOC101500209 | LOC101501730 | None |
| 4 | 11724079 | + | 5 | 32966992 | + | LOC101500209 | LOC101494179 | None |
| 4 | 11724100 | + | 7 | 1701549 | - | LOC101500209 | LOC101501730 | None |
| 4 | 11724100 | + | 7 | 19767875 | - | LOC101500209 | LOC101508402 | None |
| 4 | 11724136 | + | 6 | 58265166 | + | LOC101500209 | LOC101506890 | None |
| 4 | 12293362 | + | 7 | 9049790 | - | LOC101492195 | LOC101502286 | None |
| 4 | 13765589 | + | 8 | 617853 | - | LOC101493738 | LOC101511091 | Exon_None |
| 4 | 14069724 | + | 4 | 14083803 | - | LOC101507074 | LOC101506406 | None |
| 4 | 14069750 | - | 4 | 14088744 | - | LOC101507074 | LOC113786294 | None |
| 4 | 15076791 | + | 4 | 15070272 | + | LOC101515546 | LOC101515223 | None |
| 4 | 15076792 | + | 4 | 15070273 | + | LOC101515546 | LOC101515223 | None |
| 4 | 15076793 | + | 4 | 15070274 | + | LOC101515546 | LOC101515223 | None |
| 4 | 15076794 | + | 4 | 15070275 | + | LOC101515546 | LOC101515223 | None |
| 4 | 15076796 | + | 4 | 15070277 | + | LOC101515546 | LOC101515223 | None |
| 4 | 1561884 | - | 6 | 58273105 | + | LOC101513777 | LOC101507444 | None |
| 1 | 186719 | + | 1 | 46532096 | - | LOC101494203 | LOC101497567 | Both |
| 4 | 16278431 | - | 3 | 34452360 | - | LOC101499877 | LOC101508956 | None |
| 4 | 19020149 | + | 3 | 26295288 | - | LOC105851910 | LOC101493610 | None |
| 4 | 19020392 | + | 4 | 43479567 | - | LOC105851910 | LOC101507919 | None |
| 4 | 22375531 | - | 2 | 36191301 | - | LOC101489622 | LOC101514326 | None |
| 4 | 25254569 | - | 4 | 25286975 | - | LOC101508452 | LOC101508756 | Both |
| 4 | 2556967 | - | 1 | 10690149 | + | LOC101494047 | LOC101500372 | None |
| 4 | 2734712 | + | 4 | 2703638 | + | LOC101514244 | LOC101513258 | Exon_None |
| 4 | 30334555 | + | 3 | 5554464 | - | LOC101509942 | LOC101511513 | None |
| 4 | 30334557 | + | 3 | 5554462 | - | LOC101509942 | LOC101511513 | None |
| 4 | 31553202 | + | 6 | 10453276 | + | LOC101493097 | LOC101515132 | None |
| 1 | 22131631 | - | NW_004516527.1 | 525194 | + | LOC105852588 | LOC101501045 | Both |
| 4 | 37722642 | + | 3 | 35294187 | - | LOC101496964 | LOC101515087 | None |
| 4 | 3793903 | + | 4 | 3765761 | + | LOC101501044 | LOC101500197 | None |
| 4 | 38611709 | - | 8 | 61086 | + | LOC101495550 | LOC101512264 | Both |
| 4 | 38830108 | + | 2 | 9727681 | - | LOC101499888 | LOC101494958 | Both |
| 4 | 40168610 | - | 4 | 40204933 | - | LOC101501181 | LOC101499889 | Exon_None |
| 4 | 43441474 | - | 3 | 27878275 | - | LOC113786305 | LOC101499238 | None |
| 4 | 43891185 | + | 2 | 34245685 | + | LOC101494477 | LOC101506259 | None |
| 4 | 46430084 | + | 5 | 36666746 | + | LOC101502456 | LOC101512740 | Both |
| 4 | 4705896 | - | 3 | 2926665 | + | LOC101513360 | LOC101493155 | Exon_None |
| 1 | 24450273 | + | 6 | 7679368 | - | LOC101490106 | LOC101489323 | Both |
| 1 | 24450273 | + | 6 | 7679791 | - | LOC101490106 | LOC101489323 | Exon_None |
| 4 | 8666491 | + | 4 | 8648457 | + | LOC101496750 | LOC101494893 | None_Exon |
| 4 | 8807367 | - | 4 | 33619832 | + | LOC101501591 | LOC113786381 | Both |
| 4 | 8807367 | - | 4 | 33620013 | + | LOC101501591 | LOC113786381 | Both |
| 4 | 887192 | + | 3 | 12123321 | + | LOC101491017 | LOC105851751 | None |
| 5 | 11997177 | + | 6 | 15450781 | - | LOC101504067 | LOC113787067 | None_Exon |
| 5 | 17855071 | - | 6 | 24426206 | - | LOC101508763 | LOC101501416 | None |
| 1 | 2538887 | + | 3 | 16534249 | + | LOC101500252 | LOC101501345 | None |
| 5 | 20844865 | - | 3 | 33764259 | + | LOC113786767 | LOC101490255 | None_Exon |
| 5 | 28200368 | - | 3 | 34452352 | - | LOC105852166 | LOC101508956 | None |
| 5 | 29232366 | - | 3 | 27403339 | - | LOC101510276 | LOC101508430 | Exon_None |
| 5 | 29989618 | - | 7 | 42739035 | - | LOC101510056 | LOC101489247 | None |
| 5 | 30675522 | - | 4 | 11452274 | + | LOC101512214 | LOC101493413 | None |
| 5 | 30675525 | - | 3 | 34452357 | - | LOC101512214 | LOC101508956 | None |
| 5 | 30675525 | - | 4 | 11452269 | + | LOC101512214 | LOC101493413 | None |
| 5 | 30675525 | - | 4 | 14427147 | - | LOC101512214 | LOC101513370 | None |
| 5 | 30675535 | - | 4 | 11452259 | + | LOC101512214 | LOC101493413 | None |
| 5 | 30675550 | - | 3 | 34452355 | - | LOC101512214 | LOC101508956 | None |
| 5 | 30675569 | - | 2 | 6381354 | + | LOC101512214 | LOC101499938 | None |
| 5 | 30778772 | + | 7 | 7379193 | + | LOC101491250 | LOC101501855 | None |
| 5 | 31137965 | + | 3 | 26294024 | - | LOC101505130 | LOC101493610 | Both |
| 5 | 31763840 | - | NW_004516357.1 | 62137 | + | LOC101503743 | LOC101505762 | None |
| 5 | 31763840 | - | NW_004516357.1 | 62269 | + | LOC101503743 | LOC101505762 | None |
| 1 | 29166793 | - | 4 | 40170427 | + | LOC101500471 | LOC101500851 | None_Exon |
| 1 | 29166793 | - | 4 | 40171855 | + | LOC101500471 | LOC101500851 | None_Exon |
| 5 | 34494770 | - | NW_004522673.1 | 5200 | - | LOC101500660 | LOC101496430 | None |
| 1 | 30270824 | - | 5 | 47196140 | + | LOC101508275 | LOC101507935 | None_Exon |
| 5 | 35496439 | + | 1 | 13567904 | - | LOC101494182 | LOC101497453 | None_Exon |
| 5 | 37640967 | - | 6 | 41564486 | + | LOC101515776 | LOC101509332 | None |
| 5 | 38267287 | - | 5 | 38249972 | + | LOC101489433 | LOC101489100 | Both |
| 5 | 38642983 | - | 5 | 24597010 | - | LOC101497628 | LOC101510928 | Both |
| 5 | 38944371 | + | 6 | 58255059 | + | LOC101509740 | LOC101506557 | None |
| 5 | 38944371 | + | 6 | 58265166 | + | LOC101509740 | LOC101506890 | None |
| 5 | 38944374 | + | 6 | 58265166 | + | LOC101509740 | LOC101506890 | None |
| 5 | 38944377 | + | 7 | 6839572 | - | LOC101509740 | LOC101506791 | None |
| 5 | 38944380 | + | 6 | 24256373 | + | LOC101509740 | LOC101490833 | None |
| 5 | 38944380 | + | 7 | 3226883 | - | LOC101509740 | LOC101510518 | None |
| 5 | 3945958 | + | 3 | 5093174 | - | LOC101509949 | LOC101503913 | Exon_None |
| 5 | 3946715 | + | 3 | 5093174 | - | LOC101509949 | LOC101503913 | None |
| 1 | 329868 | + | 6 | 2636734 | + | LOC101503647 | LOC101512325 | None |
| 5 | 41103222 | - | 2 | 258241 | - | LOC101492998 | LOC105851539 | None |
| 5 | 41103801 | - | 6 | 58265166 | + | LOC101492998 | LOC101506890 | None |
| 5 | 41701939 | + | 5 | 41677974 | + | LOC101502569 | LOC101496222 | Exon_None |
| 5 | 42648080 | + | 5 | 37837157 | - | LOC101504279 | LOC101501389 | None_Exon |
| 5 | 43132228 | - | 8 | 10728746 | - | LOC101506206 | LOC101493600 | None |
| 5 | 43570492 | - | 1 | 3009297 | + | LOC101496223 | LOC101493117 | Both |
| 5 | 43950938 | + | 5 | 43955194 | - | NAC5 | LOC101510394 | Both |
| 5 | 46794716 | + | 6 | 28581336 | - | LOC101500980 | LOC101497772 | Exon_None |
| 5 | 47146708 | + | 5 | 36427842 | - | LOC101506760 | LOC105852198 | Both |
| 5 | 47370173 | + | 7 | 8435144 | + | LOC101515446 | LOC101508805 | None |
| 5 | 47370174 | + | 7 | 8435145 | + | LOC101515446 | LOC101508805 | None |
| 5 | 48062932 | - | NW_004516931.1 | 11085 | - | LOC101514374 | LOC113784864 | Exon_None |
| 5 | 48112776 | + | 8 | 14436328 | + | LOC101488993 | LOC101494865 | Both |
| 6 | 10054855 | + | 3 | 39298057 | + | LOC101502371 | LOC101498254 | None |
| 6 | 10748835 | + | 3 | 38914715 | - | LOC101499602 | LOC113785749 | None_Exon |
| 6 | 1116231 | - | 2 | 29700202 | + | LOC101512000 | LOC101494120 | None |
| 6 | 1116240 | - | 2 | 29700202 | + | LOC101512000 | LOC101494120 | None |
| 6 | 13838946 | - | 5 | 48156966 | - | LOC113787216 | LOC105852223 | None |
| 6 | 14021890 | - | 2 | 36037990 | + | LOC101496011 | LOC101504983 | None |
| 6 | 14021988 | - | 8 | 10698267 | + | LOC101496011 | LOC101504019 | None |
| 6 | 14022019 | - | 2 | 36037861 | + | LOC101496011 | LOC101504983 | None |
| 6 | 14022099 | - | 8 | 10698267 | + | LOC101496011 | LOC101504019 | None |
| 6 | 14022212 | - | 2 | 3343305 | + | LOC101496011 | LOC101514962 | None |
| 6 | 14022288 | + | 8 | 10698275 | - | LOC101496011 | LOC101504019 | None |
| 6 | 14943853 | + | 5 | 43392474 | - | LOC101504391 | LOC101491259 | Exon_None |
| 6 | 15450536 | + | 5 | 11997177 | - | LOC113787067 | LOC101504067 | Exon_None |
| 6 | 15847340 | + | 1 | 1828762 | + | LOC101500129 | LOC113788097 | None_Exon |
| 6 | 15847345 | + | 1 | 1828767 | + | LOC101500129 | LOC113788097 | None |
| 6 | 15847371 | + | 1 | 1829056 | + | LOC101500129 | LOC113788097 | Both |
| 6 | 17031746 | + | 3 | 16534246 | + | LOC101511786 | LOC101501345 | None |
| 6 | 17738439 | + | 6 | 17714024 | + | LOC101506987 | LOC101505574 | None_Exon |
| 6 | 17738537 | + | 6 | 17714024 | + | LOC101506987 | LOC101505574 | None_Exon |
| 6 | 1933217 | + | 7 | 21874916 | + | LOC101511778 | LOC101501434 | None |
| 6 | 1933220 | + | 6 | 58265166 | + | LOC101511778 | LOC101506890 | None |
| 6 | 20505694 | + | NW_004522704.1 | 673 | + | LOC101500131 | LOC101505021 | None |
| 1 | 10206701 | - | 6 | 23492668 | + | LOC101502588 | PPI | None |
| 6 | 21322921 | + | 1 | 12639212 | + | LOC101510288 | LOC101500264 | None |
| 6 | 27103908 | + | 1 | 4378552 | - | LOC101491592 | LOC101494088 | None |
| 6 | 28685347 | - | NW_004516576.1 | 364000 | - | LOC101502579 | LOC101497514 | Both |
| 6 | 28685347 | - | NW_004517338.1 | 141098 | - | LOC101502579 | LOC113784935 | Both |
| 6 | 2879111 | - | 3 | 39298055 | + | LOC101496982 | LOC101498254 | None |
| 6 | 3194275 | + | 8 | 14437125 | + | LOC101512652 | LOC101494865 | None |
| 6 | 32293512 | - | 6 | 16703214 | + | LOC101488214 | LOC101502894 | Exon_None |
| 6 | 32411643 | + | 4 | 555463 | + | LOC101499190 | LOC105851854 | None |
| 6 | 3396106 | + | 6 | 3346993 | + | LOC101494819 | LOC101493433 | Both |
| 6 | 3396106 | + | 6 | 3347657 | + | LOC101494819 | LOC101493433 | Both |
| 6 | 34235216 | - | 6 | 34394896 | - | LOC101496683 | LOC105852351 | Exon_None |
| 6 | 35799076 | - | 1 | 12404685 | + | LOC101510832 | LOC101495045 | None |
| 6 | 40269370 | + | 3 | 21802632 | - | LOC101507625 | LOC101514108 | None_Exon |
| 6 | 41576320 | + | 6 | 55393820 | + | LOC101509648 | LOC101512439 | Exon_None |
| 6 | 41576482 | + | 6 | 55393820 | + | LOC101509648 | LOC101512439 | None |
| 6 | 41576635 | + | 6 | 55393513 | + | LOC101509648 | LOC101512439 | None |
| 6 | 41830675 | - | 5 | 16122360 | + | MT-1 | LOC101506526 | None |
| 6 | 41836517 | + | 5 | 28326709 | - | LOC101513828 | LOC101507300 | None |
| 6 | 41836517 | + | 6 | 18383064 | - | LOC101513828 | LOC105851113 | None |
| 6 | 47809225 | - | NW_004516470.1 | 127108 | + | LOC101515266 | LOC101498260 | Exon_None |
| 6 | 47857942 | - | 4 | 15371376 | - | LOC101489223 | LOC101500213 | None |
| 6 | 47938263 | + | 4 | 16278277 | - | LOC101490096 | LOC101499877 | None |
| 1 | 424988 | + | 8 | 10001662 | - | LOC101508579 | LOC101494343 | None |
| 6 | 5065900 | - | 6 | 26669649 | - | LOC101511451 | LOC101503313 | None |
| 6 | 52501653 | + | 6 | 52450784 | + | LOC101500680 | LOC101500032 | Both |
| 6 | 53304323 | + | 5 | 17861724 | - | LOC101515041 | LOC101508763 | Exon_None |
| 6 | 53654943 | + | 1 | 10973532 | - | LOC101497651 | LOC101490323 | None |
| 6 | 53857641 | + | 5 | 48063594 | - | LOC101501310 | LOC101514374 | None_Exon |
| 6 | 55393372 | - | 6 | 55496422 | + | LOC101512439 | LOC101514064 | None |
| 6 | 55412531 | + | 6 | 55393820 | + | LOC101513088 | LOC101512439 | None |
| 6 | 55412531 | + | 6 | 55393975 | + | LOC101513088 | LOC101512439 | None_Exon |
| 6 | 55451491 | - | 6 | 41575554 | + | LOC101503109 | LOC101509648 | None_Exon |
| 6 | 55508253 | - | 6 | 55393820 | + | LOC101514064 | LOC101512439 | Exon_None |
| 6 | 55528752 | - | 6 | 41575554 | + | LOC101514064 | LOC101509648 | None_Exon |
| 6 | 56568473 | - | 1 | 6226171 | + | LOC101496571 | LOC101510510 | None |
| 6 | 57508662 | + | 5 | 41585015 | + | LOC101497225 | LOC101494184 | Both |
| 6 | 579413 | - | 2 | 257454 | - | LOC101494386 | LOC105851539 | None |
| 6 | 58264602 | + | 6 | 58254689 | + | LOC101506890 | LOC101506557 | Both |
| 6 | 58832150 | - | 6 | 58844011 | + | LOC101497227 | LOC101496901 | None |
| 6 | 58832317 | - | 6 | 58844011 | + | LOC101497227 | LOC101496901 | None |
| 6 | 58844100 | - | 6 | 58832228 | + | LOC101496901 | LOC101497227 | None |
| 1 | 43851669 | - | 5 | 28200381 | - | LOC105851505 | LOC105852166 | Both |
| 6 | 7429102 | + | 5 | 35187921 | + | LOC101505566 | LOC101511040 | Exon_None |
| 6 | 7603273 | + | NW_004516678.1 | 50285 | + | LOC101512656 | LOC105851297 | Exon_None |
| 6 | 7667437 | - | 5 | 46767758 | + | LOC101515781 | LOC101493226 | Both |
| 6 | 9262608 | + | 3 | 38015939 | - | LOC101499182 | LOC101494664 | Exon_None |
| 6 | 9405674 | + | 1 | 6919972 | + | LOC101504938 | LOC101512978 | None |
| 6 | 9405675 | + | 1 | 6919973 | + | LOC101504938 | LOC101512978 | None |
| 6 | 9405675 | + | 6 | 5623050 | + | LOC101504938 | LOC101515578 | None |
| 6 | 9405680 | + | 6 | 5623055 | + | LOC101504938 | LOC101515578 | None |
| 7 | 11772930 | - | 7 | 8721619 | - | LOC101488682 | LOC101489466 | Both |
| 7 | 1245565 | - | 7 | 1246950 | + | LOC101509445 | LOC101509981 | Both |
| 7 | 1245565 | - | 7 | 1247011 | + | LOC101509445 | LOC101509981 | Exon_None |
| 1 | 45244313 | - | 2 | 4797206 | - | LOC101500028 | LOC105851688 | None |
| 1 | 46720294 | - | NW_004522260.1 | 732 | + | LOC101506473 | LOC101495229 | None |
| 7 | 16268441 | + | 2 | 32140253 | - | LOC101501743 | LOC101489157 | Both |
| 7 | 17037466 | - | 3 | 27989127 | - | LOC101500904 | LOC101502944 | Exon_None |
| 7 | 17037467 | - | 3 | 27989128 | - | LOC101500904 | LOC101502944 | Exon_None |
| 7 | 17759738 | + | NW_004518291.1 | 4602 | + | LOC101495277 | LOC101498938 | None |
| 7 | 17781661 | + | 7 | 8174506 | - | LOC101496917 | LOC101507543 | None |
| 7 | 17781663 | + | 7 | 8174504 | - | LOC101496917 | LOC101507543 | None |
| 7 | 17922471 | + | 7 | 17885841 | + | LOC105852505 | LOC101498120 | None_Exon |
| 7 | 23589400 | - | 1 | 14725718 | + | LOC101489239 | LOC101506669 | None_Exon |
| 7 | 23592181 | - | 1 | 14725718 | + | LOC101489239 | LOC101506669 | Both |
| 7 | 23896324 | - | 3 | 3361909 | - | LOC101494954 | LOC101498567 | Both |
| 7 | 27323257 | + | 4 | 40096167 | + | LOC113787737 | LOC101502451 | None_Exon |
| 7 | 27633096 | - | 7 | 47988425 | + | LOC101515613 | LOC113787786 | Exon_None |
| 7 | 28703435 | + | NW_004516357.1 | 596140 | - | LOC101509032 | LOC101495642 | Exon_None |
| 7 | 28718346 | + | 7 | 28705696 | + | LOC101509032 | LOC101509032 | None |
| 7 | 35870747 | + | 5 | 30287198 | + | LOC101512901 | LOC101493645 | Both |
| 7 | 42837004 | + | 2 | 30757226 | + | LOC101511501 | LOC105851662 | None_Exon |
| 7 | 44543760 | + | 6 | 31823532 | + | LOC101511606 | LOC101494302 | None |
| 7 | 47696106 | - | 7 | 48100876 | + | LOC113787790 | LOC101499448 | None |
| 7 | 6057982 | + | 1 | 9779031 | + | LOC101493571 | LOC101511796 | None |
| 7 | 6469505 | + | 1 | 597182 | - | LOC101494942 | LOC101499917 | Both |
| 7 | 8327542 | - | 7 | 8343458 | - | LOC101497235 | LOC101498113 | Exon_None |
| 1 | 5442950 | - | 1 | 5199218 | + | LOC101501306 | LOC101494709 | None_Exon |
| 7 | 9050005 | - | NW_004515657.1 | 598234 | + | LOC101502286 | LOC101500824 | Exon_None |
| 7 | 9050007 | - | NW_004515657.1 | 598232 | + | LOC101502286 | LOC101500824 | None |
| 7 | 9050011 | - | 4 | 16583380 | - | LOC101502286 | LOC101508129 | None |
| 8 | 10727989 | - | 6 | 1325083 | - | LOC101493600 | LOC101490946 | Exon_None |
| 8 | 10728637 | - | 6 | 1325083 | - | LOC101493600 | LOC101490946 | Exon_None |
| 8 | 10887373 | + | NW_004516575.1 | 1168 | - | LOC113783912 | LOC113784679 | None |
| 8 | 14152990 | + | 7 | 44465346 | + | LOC101514104 | ARF23 | Both |
| 8 | 1433611 | + | 4 | 42774451 | - | LOC101506698 | LOC101492090 | None |
| 8 | 14437054 | + | 8 | 14760891 | + | LOC101494865 | LOC101506927 | None |
| 8 | 14693307 | + | 1 | 1944443 | - | LOC101503700 | LOC101499913 | None |
| 8 | 14984074 | - | 8 | 15002360 | + | LOC101513875 | LOC101514219 | Exon_None |
| 8 | 14984075 | - | 8 | 15002359 | + | LOC101513875 | LOC101514219 | Exon_None |
| 8 | 14984076 | - | 8 | 15002358 | + | LOC101513875 | LOC101514219 | None |
| 8 | 14986340 | - | 8 | 15000223 | + | LOC101513875 | LOC101514219 | None |
| 8 | 15000223 | - | 8 | 14986340 | + | LOC101514219 | LOC101513875 | None |
| 8 | 15002386 | + | 8 | 14984057 | - | LOC101514219 | LOC101513875 | None |
| 8 | 2588 | - | 1 | 958 | - | LOC113788070 | LOC101497325 | None |
| 8 | 618156 | - | 4 | 13765525 | + | LOC101511091 | LOC101493738 | Both |
| 8 | 6990029 | - | 8 | 2539703 | - | LOC101509048 | LOC101509472 | None |
| 8 | 7665123 | - | 6 | 23492616 | + | LOC101506265 | PPI | Exon_None |
| 8 | 7760446 | + | 3 | 34452348 | - | LOC101514329 | LOC101508956 | None |
| 8 | 8011232 | - | 2 | 1705884 | - | LOC101503032 | LOC101503341 | None |
| 8 | 957294 | + | 8 | 938147 | + | LOC105852659 | CHS | None_Exon |
| NW_004515686.1 | 321646 | - | 4 | 40170427 | + | LOC101504675 | LOC101500851 | Both |
| NW_004515716.1 | 19253 | - | 8 | 14296154 | - | LOC113783989 | LOC101489263 | Both |
| NW_004515735.1 | 195451 | + | NW_004517768.1 | 10168 | + | LOC101491642 | LOC101500529 | None_Exon |
| 1 | 6125834 | - | 3 | 14473738 | + | LOC101495690 | LOC101499232 | None |
| NW_004515837.1 | 203634 | - | 5 | 44002598 | - | LOC101488602 | LOC101510937 | Both |
| NW_004515843.1 | 20075 | - | NW_004522746.1 | 13860 | - | LOC101503481 | LOC101494793 | None |
| NW_004515920.1 | 43341 | - | 2 | 32140253 | - | LOC101490575 | LOC101489157 | Both |
| NW_004515953.1 | 37475 | + | 1 | 29617784 | + | LOC101505196 | LOC105852820 | Both |
| NW_004515983.1 | 21959 | + | 6 | 49219136 | - | LOC101511004 | LOC101504406 | Both |
| NW_004516029.1 | 234571 | - | 3 | 37683057 | + | LOC101490259 | LOC113785842 | Both |
| NW_004516029.1 | 234597 | - | 3 | 37682777 | + | LOC101490259 | LOC113785842 | None |
| NW_004516230.1 | 128700 | + | 1 | 5843228 | - | LOC101507060 | LOC101514175 | None |
| NW_004516230.1 | 128701 | + | 1 | 5843227 | - | LOC101507060 | LOC101514175 | None |
| NW_004516243.1 | 14103 | + | 3 | 452967 | - | LOC101505203 | LOC101491197 | Both |
| NW_004516243.1 | 14103 | + | 3 | 453768 | - | LOC101505203 | LOC101491197 | Exon_None |
| NW_004516264.1 | 262580 | - | 6 | 55393820 | + | LOC113784532 | LOC101512439 | None |
| NW_004516329.1 | 553399 | + | 6 | 13839022 | - | LOC101514245 | LOC113787216 | None_Exon |
| NW_004516410.1 | 3136 | + | NW_004516678.1 | 50285 | + | LOC101490268 | LOC105851297 | None |
| NW_004516410.1 | 34838 | + | NW_004516313.1 | 80977 | + | LOC113784622 | LOC101492297 | Exon_None |
| 1 | 6781654 | - | 6 | 58255086 | + | LOC101506661 | LOC101506557 | None |
| NW_004516627.1 | 74668 | - | 6 | 41575554 | + | LOC101491443 | LOC101509648 | Both |
| NW_004516695.1 | 34624 | + | NW_004516047.1 | 253087 | - | LOC101506617 | LOC101502632 | None_Exon |
| NW_004516723.1 | 109569 | + | 1 | 47888433 | + | LOC101514127 | LOC101497901 | None |
| NW_004516753.1 | 210242 | + | 7 | 13482044 | - | LOC101497726 | LOC101508599 | Both |
| NW_004516931.1 | 10722 | - | 7 | 2361041 | + | LOC113784864 | LOC101498781 | Both |
| NW_004517596.1 | 1457 | - | 5 | 20745107 | - | LOC101506738 | LOC101510273 | None_Exon |
| NW_004517817.1 | 17538 | - | NW_004516931.1 | 11085 | - | LOC101505873 | LOC113784864 | None |
| NW_004517817.1 | 17538 | - | NW_004516931.1 | 9191 | - | LOC101505873 | LOC113784864 | None_Exon |
| NW_004517947.1 | 469 | - | NW_004516545.1 | 96922 | - | LOC101489294 | LOC101494367 | None |
| NW_004517947.1 | 469 | - | NW_004516545.1 | 96926 | - | LOC101489294 | LOC101494367 | None |
| NW_004522059.1 | 377 | - | NW_004517335.1 | 55733 | - | LOC105851490 | LOC101500331 | Exon_None |
| NW_004522111.1 | 182 | - | 1 | 2855394 | - | LOC101514580 | LOC101512435 | None |
| NW_004522260.1 | 1173 | + | NW_004521997.1 | 108 | + | LOC101495229 | LOC101508351 | None_Exon |
| NW_004522260.1 | 1226 | + | NW_004521997.1 | 108 | + | LOC101495229 | LOC101508351 | None_Exon |
| NW_004522636.1 | 1764 | - | 7 | 241412 | - | LOC101491565 | LOC101505476 | Both |
| NW_004522673.1 | 467 | - | 5 | 34488433 | - | LOC101496101 | LOC101510715 | None |
| NW_004522673.1 | 471 | - | 5 | 34488437 | - | LOC101496101 | LOC101510715 | None_Exon |
| NW_004522681.1 | 340 | - | 6 | 6273742 | + | LOC101498403 | LOC101515780 | None |
| NW_004522681.1 | 340 | - | 6 | 6552461 | - | LOC101498403 | LOC101495245 | None |
| NW_004522716.1 | 5841 | + | 2 | 10219184 | - | LOC101509074 | LOC101508407 | Exon_None |
| NW_004522716.1 | 5841 | + | 2 | 10219188 | - | LOC101509074 | LOC101508407 | Exon_None |
| NW_004522716.1 | 6046 | + | 2 | 10219184 | - | LOC101509074 | LOC101508407 | Exon_None |
| NW_004522716.1 | 6046 | + | 2 | 10219188 | - | LOC101509074 | LOC101508407 | Exon_None |
| NW_004522742.1 | 426 | - | NW_004520126.1 | 9840 | - | LOC101492545 | LOC101510150 | None |
| 1 | 8839590 | - | 3 | 26933074 | - | LOC101496895 | LOC101491644 | None |
| 4 | 141545 | - | 2 | 1494384 | - | LOC101496413 | LOC101497135 | Both |
| 4 | 141545 | - | 2 | 1494384 | - | LOC101492414 | LOC101497135 | Both |

Example of fusion gene pairs having multiple fusion isoforms

| **Gene 1** | **Gene 2** | **No. of fusion isoforms** |
| --- | --- | --- |
| LOC101515546 | LOC101515223 | 5 |
| LOC101514962 | LOC101496011 | 4 |
| LOC101513875 | LOC101514219 | 4 |
| LOC101509074 | LOC101508407 | 4 |
| LOC101512687 | LOC101503191 | 3 |
| LOC101512214 | LOC101493413 | 3 |
| LOC101509648 | LOC101512439 | 3 |
| LOC101500129 | LOC113788097 | 3 |
| LOC101496011 | LOC101504019 | 3 |
| LOC105851539 | LOC101512000 | 2 |

Example of genes involved in multiple fusion events

| **Gene 1** | **Gene 2** | **No. of fusion gene partners** |
| --- | --- | --- |
| LOC101500209 | LOC101506557, LOC101491644, LOC101501730, LOC101494179, LOC101501730, LOC101508402, LOC101506890 | 7 |
| LOC101509740 | LOC101506557, LOC101506890, LOC101506890, LOC101506791, LOC101490833, LOC101510518 | 6 |
| LOC101502810 | LOC101511778, LOC101507074, LOC101493413, LOC101494085, LOC101494711, LOC101495739 | 6 |
| LOC101496011 | LOC101514962, LOC101499819, LOC101504983, LOC101504019 | 4 |

Example of fusion gene pairs with unique partner

| **Gene 1** | **Gene 2** |
| --- | --- |
| LOC101488214 | LOC101502894 |
| LOC101491737 | LOC101494711 |
| LOC101494203 | LOC101497567 |
| LOC101495550 | LOC101512264 |
