# Supplementary material for: Molecular and expression analyses indicate the role of fusion transcripts in mediating abiotic stress responses in chickpea
Source: Front Plant Sci. 2025 Oct 31;16:1677098. doi: 10.3389/fpls.2025.1677098 (PMC12615446; doi:10.3389/fpls.2025.1677098)
Supplement: Supplementary Table 4 — Information regarding Short homologous sequences (SHSs) detected at the junction of the fusion transcript. [file Table4.docx]

**Table S4.** Information regarding Short homologous sequences (SHSs) detected at the junction of fusion transcript.

| **Coordinate 1** | **Coordinate 2** | **Splice pattern** | **SHSs** | **Length of SHS** |
| --- | --- | --- | --- | --- |
| 2:247415-247419(+) | 2:29700239-29700243(+) | GCCU | GCCT | 4 |
| 2:257064-257068(+) | 2:29700239-29700243(+) | GCCU | TTGAACAGCCT | 11 |
| 2:30028514-30028518(+) | 4:11451524-11451528(+) | AUGU | CATGT | 5 |
| 2:30144896-30144900(-) | 4:2023968-2023972(+) | GAAG | GGAAGAG | 7 |
| 3:28995360-28995364(-) | 3:29007308-29007312(-) | ACAU | ACAT | 4 |
| 1:16466252-16466256(+) | 6:14021846-14021850(-) | AUGU | CTATGTTT | 6 |
| 4:15076794-15076798(+) | 4:15070274-15070278(+) | AUAU | TATATTCA | 8 |
| 6:15847343-15847347(+) | 1:1828764-1828768(+) | AUGU | GGATGTCCATACCAGAT | 17 |
| 6:3194273-3194277(+) | 8:14437122-14437126(+) | AUGU | GATGT | 5 |
| 6:41576633-41576637(+) | 6:55393510-55393514(+) | AUGU | AAATGTTA | 6 |
| 6:55393369-55393373(-) | 6:55496419-55496423(+) | ACAU | ACAT | 4 |
| 8:14437052-14437056(+) | 8:14760888-14760892(+) | AGGC | AGGCC | 5 |
| 8:14984071-14984075(-) | 8:15002357-15002361(+) | AACA | AATACAAA | 8 |
| 8:14986337-14986341(-) | 8:15000220-15000224(+) | ACCU | ACCT | 4 |
| 2:247456-247464(-) | 2:257534-257542(+) | GUAG | GAAGGTA | 7 |
| 2:25534566-25534574(-) | 7:22344916-22344924(-) | GUAG | ATAGGTGC | 8 |
| 2:7668378-7668386(-) | 4:11451535-11451543(+) | GUAG | TAGGTG | 6 |
| 4:11724042-11724050(+) | 3:26933070-26933078(-) | GUAG | TGGAGGTGAG | 10 |
| 4:11724075-11724083(+) | 5:32966987-32966995 | GUAG | GAAGGTGA | 8 |
| 4:11724096-11724104(+) | 7:1701545-1701553(-) | GUAG | GAAGGTGG | 8 |
| 4:11724096-11724104(+) | 7:19767871-19767879(-) | GUAG | GGAGGT | 6 |
| 4:14069745-14069753(-) | 4:14088740-14088748(-) | GUAG | AGGTTG | 6 |
| 4:19020145-19020153(+) | 3:26295284-26295292(-) | GUAG | GGAGGTG | 7 |
| 4:43441469-43441477(-) | 3:27878271-27878279(-) | GUAG | GAGGTG | 6 |
| 4:43891181-43891189(+) | 2:34245680-34245688(+) | GUAG | GAAGGTT | 7 |
| 5:29989613-29989621(-) | 7:42739031-42739039(-) | GUAG | AAAGGT | 6 |
| 6:14021885-14021893(-) | 2:36037985-36037993(+) | GUAG | GAAGGTGA | 8 |
| 6:14022207-14022215(-) | 2:3343300-3343308(+) | GUAG | CAGGTG | 6 |
| 6:41836513-41836521(+) | 5:28326705-28326713(-) | GUAG | AGAGGTC | 7 |
| 6:47938259-47938267(+) | 4:16278273-16278281(-) | GUAG | GAAGGT | 6 |
| 1:424984-424992(+) | 8:10001658-10001666 | GUAG | GGAGGTGC | 8 |
| 7:44543756-44543764(+) | 6:31823527-31823535(+) | GUAG | CAGAGGTGTA | 10 |
| 8:15000218-15000226(-) | 8:14986335-14986343(+) | GUAG | AGAGGTGT | 8 |
