# Supplementary material for: Molecular and expression analyses indicate the role of fusion transcripts in mediating abiotic stress responses in chickpea
Source: Front Plant Sci. 2025 Oct 31;16:1677098. doi: 10.3389/fpls.2025.1677098 (PMC12615446; doi:10.3389/fpls.2025.1677098)
Supplement: Supplementary Table 5 — Predicted coding potential of fusion transcripts. [file Table5.docx]

**Table S5.** Predicted coding potential of fusion transcripts.

| **Fusion transcripts** | **CPAT Prediction** | **PlncPro Prediction** | **CNIT Prediction** | **Probable annotations** |
| --- | --- | --- | --- | --- |
| >2:2062656_2062857_3:36308937_36309138 | Non-Coding | Non-Coding | Non-Coding | Non-Coding |
| >2:23931140_23931340_NW_004517187.1:5630_5831 | Coding | Non-Coding | Non-Coding | Non-Coding |
| >2:23933374_23933574_NW_004517187.1:5630_5831 | Non-Coding | Non-Coding | Non-Coding | Non-Coding |
| >2:23933794_23933994_NW_004517187.1:5630_5831 | Coding | Non-Coding | Non-Coding | Non-Coding |
| >2:247217_247417_2:29700241_29700442 | Coding | Non-Coding | Non-Coding | Non-Coding |
| >2:247217_247417_6:1116101_1116301 | Coding | Non-Coding | Non-Coding | Non-Coding |
| >2:247460_247661_2:257538_257739 | Coding | Non-Coding | Non-Coding | Non-Coding |
| >2:25534570_25534771_7:22344720_22344920 | Coding | Non-Coding | Non-Coding | Non-Coding |
| >2:256848_257048_6:1116119_1116319 | Coding | Non-Coding | Non-Coding | Non-Coding |
| >2:256866_257066_2:29700241_29700442 | Coding | Non-Coding | Non-Coding | Non-Coding |
| >2:256866_257066_6:1116101_1116301 | Coding | Non-Coding | Non-Coding | Non-Coding |
| >2:30028316_30028516_4:11451526_11451727 | Coding | Non-Coding | Non-Coding | Non-Coding |
| >2:30144898_30145099_4:2023970_2024171 | Coding | Coding | Non-Coding | Coding |
| >2:30744588_30744789_7:13554956_13555156 | Non-Coding | Non-Coding | Non-Coding | Non-Coding |
| >2:30965638_30965839_3:34452152_34452352 | Coding | Non-Coding | Non-Coding | Non-Coding |
| >2:31356743_31356943_3:39263829_39264029 | Coding | Non-Coding | Non-Coding | Non-Coding |
| >2:31357989_31358189_3:21634829_21635029 | Coding | Non-Coding | Non-Coding | Non-Coding |
| >2:31357989_31358189_3:21635386_21635586 | Coding | Non-Coding | Non-Coding | Non-Coding |
| >2:3343312_3343513_6:14022202_14022403 | Coding | Non-Coding | Non-Coding | Non-Coding |
| >2:3343175_3343375_6:14022105_14022305 | Coding | Non-Coding | Non-Coding | Non-Coding |
| >2:3343693_3343894_6:14021907_14022108 | Coding | Non-Coding | Non-Coding | Non-Coding |
| >2:3343511_3343711_6:14021689_14021889 | Coding | Non-Coding | Non-Coding | Non-Coding |
| >1:13497108_13497308_2:30479153_30479354 | Coding | Non-Coding | Non-Coding | Non-Coding |
| >2:34908511_34908711_2:34943518_34943719 | Coding | Non-Coding | Non-Coding | Non-Coding |
| >2:35441073_35441273_4:40170426_40170627 | Non-Coding | Non-Coding | Non-Coding | Non-Coding |
| >2:36050972_36051172_2:3642670_3642870 | Coding | Non-Coding | Non-Coding | Non-Coding |
| >2:6229424_6229625_1:32602222_32602422 | Coding | Non-Coding | Non-Coding | Non-Coding |
| >2:6381126_6381327_5:45803310_45803511 | Coding | Non-Coding | Non-Coding | Non-Coding |
| >2:6381028_6381228_2:36191205_36191405 | Coding | Non-Coding | Coding | Coding |
| >2:6413831_6414031_7:13670916_13671117 | Coding | Non-Coding | Non-Coding | Non-Coding |
| >2:7569150_7569350_7:18818098_18818298 | Coding | Non-Coding | Non-Coding | Non-Coding |
| >2:7668306_7668507_6:1932446_1932646 | Coding | Non-Coding | Non-Coding | Non-Coding |
| >2:7668364_7668565_4:14069697_14069897 | Coding | Coding | Non-Coding | Coding |
| >2:7668382_7668583_4:11451539_11451740 | Non-Coding | Non-Coding | Non-Coding | Non-Coding |
| >2:7668386_7668587_6:19725942_19726142 | Coding | Non-Coding | Non-Coding | Non-Coding |
| >2:7668503_7668704_1:6097476_6097677 | Coding | Coding | Non-Coding | Coding |
| >2:7668517_7668718_8:13248133_13248333 | Coding | Non-Coding | Non-Coding | Non-Coding |
| >2:8273512_8273713_6:9515612_9515813 | Coding | Non-Coding | Non-Coding | Non-Coding |
| >2:8294350_8294551_6:23393184_23393385 | Coding | Non-Coding | Non-Coding | Non-Coding |
| >2:8295602_8295803_6:23393178_23393379 | Coding | Non-Coding | Non-Coding | Non-Coding |
| >3:16533316_16533516_1:2538835_2539036 | Coding | Coding | Coding | Coding |
| >3:23900663_23900864_2:8516422_8516622 | Coding | Non-Coding | Non-Coding | Non-Coding |
| >3:26932907_26933108_5:33690950_33691151 | Coding | Coding | Coding | Coding |
| >3:27404163_27404363_8:5439099_5439300 | Coding | Non-Coding | Non-Coding | Non-Coding |
| >3:27784878_27785079_3:34452157_34452357 | Coding | Non-Coding | Non-Coding | Non-Coding |
| >3:27845437_27845638_2:27608495_27608696 | Coding | Non-Coding | Non-Coding | Non-Coding |
| >3:27956776_27956976_3:31303422_31303623 | Coding | Non-Coding | Non-Coding | Non-Coding |
| >3:28523280_28523480_7:40734251_40734451 | Coding | Non-Coding | Non-Coding | Non-Coding |
| >3:2879882_2880083_1:6097563_6097764 | Coding | Non-Coding | Coding | Coding |
| >1:16466008_16466208_6:14021694_14021894 | Coding | Non-Coding | Non-Coding | Non-Coding |
| >3:28995362_28995563_3:29007110_29007310 | Coding | Non-Coding | Non-Coding | Non-Coding |
| >3:29226106_29226306_2:24651695_24651896 | Coding | Non-Coding | Non-Coding | Non-Coding |
| >1:16466054_16466254_6:14021648_14021848 | Coding | Non-Coding | Non-Coding | Non-Coding |
| >3:31623091_31623291_2:31359282_31359483 | Coding | Non-Coding | Non-Coding | Non-Coding |
| >3:32961387_32961588_6:19735633_19735834 | Coding | Non-Coding | Non-Coding | Non-Coding |
| >3:34502646_34502846_NW_004522750.1:16873_17073 | Non-Coding | Non-Coding | Non-Coding | Non-Coding |
| >3:35829659_35829859_4:40170426_40170627 | Non-Coding | Non-Coding | Coding | Non-Coding |
| >3:36712101_36712301_4:43532655_43532856 | Coding | Non-Coding | Non-Coding | Non-Coding |
| >3:36712101_36712301_6:32237812_32238013 | Coding | Non-Coding | Non-Coding | Non-Coding |
| >3:37682893_37683093_NW_004516029.1:233817_234017 | Coding | Non-Coding | Non-Coding | Non-Coding |
| >3:39340161_39340361_3:39300068_39300269 | Coding | Non-Coding | Non-Coding | Non-Coding |
| >3:39751661_39751862_5:30675309_30675509 | Coding | Non-Coding | Non-Coding | Non-Coding |
| >3:39988335_39988535_1:758_958 | Coding | Non-Coding | Non-Coding | Non-Coding |
| >3:6965532_6965732_3:6838468_6838668 | Coding | Non-Coding | Non-Coding | Non-Coding |
| >3:974127_974327_1:3266648_3266849 | Coding | Coding | Non-Coding | Coding |
| >4:11377975_11378175_4:45752818_45753019 | Coding | Non-Coding | Non-Coding | Non-Coding |
| >4:11723807_11724007_6:58255083_58255284 | Coding | Non-Coding | Non-Coding | Non-Coding |
| >4:11723846_11724046_3:26932874_26933074 | Coding | Coding | Non-Coding | Coding |
| >4:11723846_11724046_7:1701409_1701609 | Coding | Non-Coding | Non-Coding | Non-Coding |
| >4:11723879_11724079_5:32966991_32967192 | Coding | Non-Coding | Non-Coding | Non-Coding |
| >4:11723900_11724100_7:1701349_1701549 | Coding | Non-Coding | Non-Coding | Non-Coding |
| >4:11723900_11724100_7:19767675_19767875 | Coding | Non-Coding | Non-Coding | Non-Coding |
| >4:11723936_11724136_6:58265165_58265366 | Coding | Coding | Non-Coding | Coding |
| >4:12293162_12293362_7:9049590_9049790 | Coding | Non-Coding | Non-Coding | Non-Coding |
| >4:13765389_13765589_8:617653_617853 | Non-Coding | Non-Coding | Non-Coding | Non-Coding |
| >4:14069524_14069724_4:14083603_14083803 | Non-Coding | Non-Coding | Non-Coding | Non-Coding |
| >4:14069749_14069950_4:14088544_14088744 | Coding | Non-Coding | Non-Coding | Non-Coding |
| >4:15076591_15076791_4:15070271_15070472 | Coding | Non-Coding | Non-Coding | Non-Coding |
| >4:15076592_15076792_4:15070272_15070473 | Coding | Non-Coding | Non-Coding | Non-Coding |
| >4:15076593_15076793_4:15070273_15070474 | Coding | Non-Coding | Non-Coding | Non-Coding |
| >4:15076594_15076794_4:15070274_15070475 | Coding | Non-Coding | Non-Coding | Non-Coding |
| >4:15076596_15076796_4:15070276_15070477 | Coding | Non-Coding | Non-Coding | Non-Coding |
| >4:1561883_1562084_6:58273104_58273305 | Coding | Non-Coding | Non-Coding | Non-Coding |
| >1:186519_186719_1:46531896_46532096 | Coding | Non-Coding | Non-Coding | Non-Coding |
| >4:16278430_16278631_3:34452160_34452360 | Coding | Non-Coding | Non-Coding | Non-Coding |
| >4:19019949_19020149_3:26295088_26295288 | Coding | Non-Coding | Non-Coding | Non-Coding |
| >4:19020192_19020392_4:43479367_43479567 | Coding | Coding | Non-Coding | Coding |
| >4:22375530_22375731_2:36191101_36191301 | Coding | Non-Coding | Non-Coding | Non-Coding |
| >4:25254568_25254769_4:25286775_25286975 | Coding | Non-Coding | Coding | Coding |
| >4:2556966_2557167_1:10690148_10690349 | Coding | Non-Coding | Coding | Coding |
| >4:2734512_2734712_4:2703637_2703838 | Coding | Non-Coding | Non-Coding | Non-Coding |
| >4:30334355_30334555_3:5554264_5554464 | Coding | Non-Coding | Non-Coding | Non-Coding |
| >4:30334357_30334557_3:5554262_5554462 | Coding | Non-Coding | Non-Coding | Non-Coding |
| >4:31553002_31553202_6:10453275_10453476 | Coding | Non-Coding | Non-Coding | Non-Coding |
| >1:22131630_22131831_NW_004516527.1:525193_525394 | Non-Coding | Non-Coding | Non-Coding | Non-Coding |
| >4:37722442_37722642_3:35293987_35294187 | Coding | Non-Coding | Non-Coding | Non-Coding |
| >4:3793703_3793903_4:3765760_3765961 | Coding | Non-Coding | Non-Coding | Non-Coding |
| >4:38611708_38611909_8:61085_61286 | Coding | Non-Coding | Non-Coding | Non-Coding |
| >4:38829908_38830108_2:9727481_9727681 | Coding | Non-Coding | Non-Coding | Non-Coding |
| >4:40168609_40168810_4:40204733_40204933 | Coding | Non-Coding | Coding | Coding |
| >4:43441473_43441674_3:27878075_27878275 | Coding | Non-Coding | Non-Coding | Non-Coding |
| >4:43890985_43891185_2:34245684_34245885 | Coding | Non-Coding | Non-Coding | Non-Coding |
| >4:46429884_46430084_5:36666745_36666946 | Coding | Non-Coding | Non-Coding | Non-Coding |
| >4:4705895_4706096_3:2926664_2926865 | Coding | Non-Coding | Non-Coding | Non-Coding |
| >1:24450073_24450273_6:7679168_7679368 | Coding | Non-Coding | Non-Coding | Non-Coding |
| >1:24450073_24450273_6:7679591_7679791 | Coding | Non-Coding | Coding | Coding |
| >4:8666291_8666491_4:8648456_8648657 | Coding | Non-Coding | Coding | Coding |
| >4:8807366_8807567_4:33619831_33620032 | Non-Coding | Non-Coding | Non-Coding | Non-Coding |
| >4:8807366_8807567_4:33620012_33620213 | Coding | Non-Coding | Non-Coding | Non-Coding |
| >4:886992_887192_3:12123320_12123521 | Coding | Non-Coding | Non-Coding | Non-Coding |
| >5:11996977_11997177_6:15450581_15450781 | Coding | Non-Coding | Non-Coding | Non-Coding |
| >5:17855070_17855271_6:24426006_24426206 | Coding | Non-Coding | Non-Coding | Non-Coding |
| >1:2538687_2538887_3:16534248_16534449 | Coding | Non-Coding | Non-Coding | Non-Coding |
| >5:20844864_20845065_3:33764258_33764459 | Non-Coding | Non-Coding | Non-Coding | Non-Coding |
| >5:28200367_28200568_3:34452152_34452352 | Coding | Non-Coding | Non-Coding | Non-Coding |
| >5:29232365_29232566_3:27403139_27403339 | Coding | Non-Coding | Non-Coding | Non-Coding |
| >5:29989617_29989818_7:42738835_42739035 | Coding | Coding | Non-Coding | Coding |
| >5:30675521_30675722_4:11452273_11452474 | Coding | Non-Coding | Non-Coding | Non-Coding |
| >5:30675524_30675725_3:34452157_34452357 | Coding | Non-Coding | Non-Coding | Non-Coding |
| >5:30675524_30675725_4:11452268_11452469 | Coding | Non-Coding | Non-Coding | Non-Coding |
| >5:30675524_30675725_4:14426947_14427147 | Coding | Non-Coding | Non-Coding | Non-Coding |
| >5:30675534_30675735_4:11452258_11452459 | Coding | Non-Coding | Non-Coding | Non-Coding |
| >5:30675549_30675750_3:34452155_34452355 | Coding | Non-Coding | Non-Coding | Non-Coding |
| >5:30675568_30675769_2:6381353_6381554 | Non-Coding | Non-Coding | Non-Coding | Non-Coding |
| >5:30778572_30778772_7:7379192_7379393 | Coding | Non-Coding | Non-Coding | Non-Coding |
| >5:31137765_31137965_3:26293824_26294024 | Coding | Non-Coding | Non-Coding | Non-Coding |
| >5:31763839_31764040_NW_004516357.1:62136_62337 | Coding | Non-Coding | Non-Coding | Non-Coding |
| >5:31763839_31764040_NW_004516357.1:62268_62469 | Coding | Coding | Non-Coding | Coding |
| >1:29166792_29166993_4:40170426_40170627 | Non-Coding | Non-Coding | Non-Coding | Non-Coding |
| >1:29166792_29166993_4:40171854_40172055 | Coding | Non-Coding | Non-Coding | Non-Coding |
| >5:34494769_34494970_NW_004522673.1:5000_5200 | Coding | Non-Coding | Non-Coding | Non-Coding |
| >1:30270823_30271024_5:47196139_47196340 | Coding | Non-Coding | Non-Coding | Non-Coding |
| >5:35496239_35496439_1:13567704_13567904 | Coding | Non-Coding | Non-Coding | Non-Coding |
| >5:37640966_37641167_6:41564485_41564686 | Coding | Non-Coding | Non-Coding | Non-Coding |
| >5:38267286_38267487_5:38249971_38250172 | Coding | Non-Coding | Non-Coding | Non-Coding |
| >5:38642982_38643183_5:24596810_24597010 | Coding | Non-Coding | Non-Coding | Non-Coding |
| >5:38944171_38944371_6:58255058_58255259 | Coding | Non-Coding | Non-Coding | Non-Coding |
| >5:38944171_38944371_6:58265165_58265366 | Coding | Non-Coding | Non-Coding | Non-Coding |
| >5:38944174_38944374_6:58265165_58265366 | Coding | Non-Coding | Non-Coding | Non-Coding |
| >5:38944177_38944377_7:6839372_6839572 | Coding | Non-Coding | Non-Coding | Non-Coding |
| >5:38944180_38944380_6:24256372_24256573 | Coding | Non-Coding | Non-Coding | Non-Coding |
| >5:38944180_38944380_7:3226683_3226883 | Coding | Non-Coding | Non-Coding | Non-Coding |
| >5:3945758_3945958_3:5092974_5093174 | Coding | Non-Coding | Non-Coding | Non-Coding |
| >5:3946515_3946715_3:5092974_5093174 | Coding | Coding | Non-Coding | Coding |
| >1:329668_329868_6:2636733_2636934 | Coding | Non-Coding | Non-Coding | Non-Coding |
| >5:41103221_41103422_2:258041_258241 | Coding | Non-Coding | Non-Coding | Non-Coding |
| >5:41103800_41104001_6:58265165_58265366 | Coding | Non-Coding | Non-Coding | Non-Coding |
| >5:41701739_41701939_5:41677973_41678174 | Coding | Non-Coding | Non-Coding | Non-Coding |
| >5:42647880_42648080_5:37836957_37837157 | Coding | Non-Coding | Non-Coding | Non-Coding |
| >5:43132227_43132428_8:10728546_10728746 | Coding | Non-Coding | Non-Coding | Non-Coding |
| >5:43570491_43570692_1:3009296_3009497 | Non-Coding | Non-Coding | Non-Coding | Non-Coding |
| >5:43950738_43950938_5:43954994_43955194 | Coding | Non-Coding | Non-Coding | Non-Coding |
| >5:46794516_46794716_6:28581136_28581336 | Coding | Non-Coding | Non-Coding | Non-Coding |
| >5:47146508_47146708_5:36427642_36427842 | Non-Coding | Non-Coding | Non-Coding | Non-Coding |
| >5:47369973_47370173_7:8435143_8435344 | Coding | Non-Coding | Non-Coding | Non-Coding |
| >5:47369974_47370174_7:8435144_8435345 | Coding | Non-Coding | Non-Coding | Non-Coding |
| >5:48062931_48063132_NW_004516931.1:10885_11085 | Coding | Non-Coding | Coding | Coding |
| >5:48112576_48112776_8:14436327_14436528 | Coding | Non-Coding | Non-Coding | Non-Coding |
| >6:10054655_10054855_3:39298056_39298257 | Coding | Non-Coding | Non-Coding | Non-Coding |
| >6:10748635_10748835_3:38914515_38914715 | Non-Coding | Non-Coding | Non-Coding | Non-Coding |
| >6:1116230_1116431_2:29700201_29700402 | Coding | Coding | Coding | Coding |
| >6:1116239_1116440_2:29700201_29700402 | Coding | Coding | Coding | Coding |
| >6:13838945_13839146_5:48156766_48156966 | Coding | Non-Coding | Non-Coding | Non-Coding |
| >6:14021889_14022090_2:36037989_36038190 | Coding | Non-Coding | Non-Coding | Non-Coding |
| >6:14021987_14022188_8:10698266_10698467 | Coding | Non-Coding | Non-Coding | Non-Coding |
| >6:14022018_14022219_2:36037860_36038061 | Coding | Coding | Non-Coding | Coding |
| >6:14022098_14022299_8:10698266_10698467 | Coding | Non-Coding | Non-Coding | Non-Coding |
| >6:14022211_14022412_2:3343304_3343505 | Coding | Non-Coding | Non-Coding | Non-Coding |
| >6:14022088_14022288_8:10698075_10698275 | Coding | Non-Coding | Non-Coding | Non-Coding |
| >6:14943653_14943853_5:43392274_43392474 | Coding | Non-Coding | Non-Coding | Non-Coding |
| >6:15450336_15450536_5:11996977_11997177 | Coding | Non-Coding | Non-Coding | Non-Coding |
| >6:15847140_15847340_1:1828761_1828962 | Coding | Non-Coding | Non-Coding | Non-Coding |
| >6:15847145_15847345_1:1828766_1828967 | Coding | Non-Coding | Non-Coding | Non-Coding |
| >6:15847171_15847371_1:1829055_1829256 | Coding | Non-Coding | Non-Coding | Non-Coding |
| >6:17031546_17031746_3:16534245_16534446 | Coding | Non-Coding | Non-Coding | Non-Coding |
| >6:17738239_17738439_6:17714023_17714224 | Coding | Non-Coding | Non-Coding | Non-Coding |
| >6:17738337_17738537_6:17714023_17714224 | Coding | Non-Coding | Non-Coding | Non-Coding |
| >6:1933017_1933217_7:21874915_21875116 | Coding | Non-Coding | Non-Coding | Non-Coding |
| >6:1933020_1933220_6:58265165_58265366 | Coding | Non-Coding | Non-Coding | Non-Coding |
| >6:20505494_20505694_NW_004522704.1:672_873 | Coding | Non-Coding | Coding | Coding |
| >1:10206700_10206901_6:23492667_23492868 | Non-Coding | Non-Coding | Non-Coding | Non-Coding |
| >6:21322721_21322921_1:12639211_12639412 | Coding | Non-Coding | Coding | Coding |
| >6:27103708_27103908_1:4378352_4378552 | Coding | Non-Coding | Non-Coding | Non-Coding |
| >6:28685346_28685547_NW_004516576.1:363800_364000 | Coding | Non-Coding | Non-Coding | Non-Coding |
| >6:28685346_28685547_NW_004517338.1:140898_141098 | Coding | Non-Coding | Non-Coding | Non-Coding |
| >6:2879110_2879311_3:39298054_39298255 | Coding | Non-Coding | Non-Coding | Non-Coding |
| >6:3194075_3194275_8:14437124_14437325 | Coding | Non-Coding | Non-Coding | Non-Coding |
| >6:32293511_32293712_6:16703213_16703414 | Coding | Non-Coding | Non-Coding | Non-Coding |
| >6:32411443_32411643_4:555462_555663 | Coding | Coding | Non-Coding | Coding |
| >6:3395906_3396106_6:3346992_3347193 | Coding | Non-Coding | Non-Coding | Non-Coding |
| >6:3395906_3396106_6:3347656_3347857 | Coding | Non-Coding | Non-Coding | Non-Coding |
| >6:34235215_34235416_6:34394696_34394896 | Coding | Non-Coding | Non-Coding | Non-Coding |
| >6:35799075_35799276_1:12404684_12404885 | Coding | Non-Coding | Non-Coding | Non-Coding |
| >6:40269170_40269370_3:21802432_21802632 | Coding | Non-Coding | Non-Coding | Non-Coding |
| >6:41576120_41576320_6:55393819_55394020 | Coding | Non-Coding | Non-Coding | Non-Coding |
| >6:41576282_41576482_6:55393819_55394020 | Coding | Non-Coding | Non-Coding | Non-Coding |
| >6:41576435_41576635_6:55393512_55393713 | Non-Coding | Non-Coding | Non-Coding | Non-Coding |
| >6:41830674_41830875_5:16122359_16122560 | Coding | Non-Coding | Non-Coding | Non-Coding |
| >6:41836317_41836517_5:28326509_28326709 | Coding | Non-Coding | Non-Coding | Non-Coding |
| >6:41836317_41836517_6:18382864_18383064 | Coding | Non-Coding | Non-Coding | Non-Coding |
| >6:47809224_47809425_NW_004516470.1:127107_127308 | Coding | Non-Coding | Non-Coding | Non-Coding |
| >6:47857941_47858142_4:15371176_15371376 | Coding | Non-Coding | Non-Coding | Non-Coding |
| >6:47938063_47938263_4:16278077_16278277 | Coding | Non-Coding | Non-Coding | Non-Coding |
| >1:424788_424988_8:10001462_10001662 | Coding | Non-Coding | Non-Coding | Non-Coding |
| >6:5065899_5066100_6:26669449_26669649 | Coding | Non-Coding | Non-Coding | Non-Coding |
| >6:52501453_52501653_6:52450783_52450984 | Coding | Non-Coding | Non-Coding | Non-Coding |
| >6:53304123_53304323_5:17861524_17861724 | Coding | Coding | Non-Coding | Coding |
| >6:53654743_53654943_1:10973332_10973532 | Coding | Non-Coding | Non-Coding | Non-Coding |
| >6:53857441_53857641_5:48063394_48063594 | Coding | Non-Coding | Non-Coding | Non-Coding |
| >6:55393371_55393572_6:55496421_55496622 | Non-Coding | Non-Coding | Non-Coding | Non-Coding |
| >6:55412331_55412531_6:55393819_55394020 | Coding | Non-Coding | Non-Coding | Non-Coding |
| >6:55412331_55412531_6:55393974_55394175 | Coding | Non-Coding | Non-Coding | Non-Coding |
| >6:55451490_55451691_6:41575553_41575754 | Non-Coding | Non-Coding | Non-Coding | Non-Coding |
| >6:55508252_55508453_6:55393819_55394020 | Coding | Non-Coding | Non-Coding | Non-Coding |
| >6:55528751_55528952_6:41575553_41575754 | Non-Coding | Non-Coding | Non-Coding | Non-Coding |
| >6:56568472_56568673_1:6226170_6226371 | Coding | Non-Coding | Non-Coding | Non-Coding |
| >6:57508462_57508662_5:41585014_41585215 | Coding | Non-Coding | Non-Coding | Non-Coding |
| >6:579412_579613_2:257254_257454 | Non-Coding | Non-Coding | Non-Coding | Non-Coding |
| >6:58264402_58264602_6:58254688_58254889 | Coding | Coding | Coding | Coding |
| >6:58832149_58832350_6:58844010_58844211 | Coding | Non-Coding | Non-Coding | Non-Coding |
| >6:58832316_58832517_6:58844010_58844211 | Coding | Non-Coding | Non-Coding | Non-Coding |
| >6:58844099_58844300_6:58832227_58832428 | Coding | Non-Coding | Non-Coding | Non-Coding |
| >1:43851668_43851869_5:28200181_28200381 | Coding | Non-Coding | Non-Coding | Non-Coding |
| >6:7428902_7429102_5:35187920_35188121 | Coding | Non-Coding | Non-Coding | Non-Coding |
| >6:7603073_7603273_NW_004516678.1:50284_50485 | Non-Coding | Non-Coding | Non-Coding | Non-Coding |
| >6:7667436_7667637_5:46767757_46767958 | Coding | Non-Coding | Non-Coding | Non-Coding |
| >6:9262408_9262608_3:38015739_38015939 | Coding | Non-Coding | Non-Coding | Non-Coding |
| >6:9405474_9405674_1:6919971_6920172 | Coding | Non-Coding | Non-Coding | Non-Coding |
| >6:9405475_9405675_1:6919972_6920173 | Coding | Non-Coding | Non-Coding | Non-Coding |
| >6:9405475_9405675_6:5623049_5623250 | Coding | Non-Coding | Coding | Coding |
| >6:9405480_9405680_6:5623054_5623255 | Coding | Non-Coding | Coding | Coding |
| >7:11772929_11773130_7:8721419_8721619 | Coding | Non-Coding | Non-Coding | Non-Coding |
| >7:1245564_1245765_7:1246949_1247150 | Coding | Non-Coding | Non-Coding | Non-Coding |
| >7:1245564_1245765_7:1247010_1247211 | Coding | Coding | Non-Coding | Coding |
| >1:45244312_45244513_2:4797006_4797206 | Coding | Non-Coding | Non-Coding | Non-Coding |
| >1:46720293_46720494_NW_004522260.1:731_932 | Coding | Non-Coding | Non-Coding | Non-Coding |
| >7:16268241_16268441_2:32140053_32140253 | Coding | Coding | Non-Coding | Coding |
| >7:17037465_17037666_3:27988927_27989127 | Coding | Non-Coding | Non-Coding | Non-Coding |
| >7:17037466_17037667_3:27988928_27989128 | Coding | Non-Coding | Non-Coding | Non-Coding |
| >7:17759538_17759738_NW_004518291.1:4601_4802 | Coding | Non-Coding | Non-Coding | Non-Coding |
| >7:17781461_17781661_7:8174306_8174506 | Coding | Non-Coding | Non-Coding | Non-Coding |
| >7:17781463_17781663_7:8174304_8174504 | Coding | Coding | Non-Coding | Coding |
| >7:17922271_17922471_7:17885840_17886041 | Coding | Non-Coding | Non-Coding | Non-Coding |
| >7:23589399_23589600_1:14725717_14725918 | Coding | Non-Coding | Non-Coding | Non-Coding |
| >7:23592180_23592381_1:14725717_14725918 | Coding | Non-Coding | Non-Coding | Non-Coding |
| >7:23896323_23896524_3:3361709_3361909 | Coding | Non-Coding | Non-Coding | Non-Coding |
| >7:27323057_27323257_4:40096166_40096367 | Coding | Non-Coding | Non-Coding | Non-Coding |
| >7:27633095_27633296_7:47988424_47988625 | Coding | Non-Coding | Non-Coding | Non-Coding |
| >7:28703235_28703435_NW_004516357.1:595940_596140 | Coding | Non-Coding | Non-Coding | Non-Coding |
| >7:28718146_28718346_7:28705695_28705896 | Coding | Non-Coding | Non-Coding | Non-Coding |
| >7:35870547_35870747_5:30287197_30287398 | Coding | Non-Coding | Coding | Coding |
| >7:42836804_42837004_2:30757225_30757426 | Coding | Non-Coding | Non-Coding | Non-Coding |
| >7:44543560_44543760_6:31823531_31823732 | Coding | Non-Coding | Non-Coding | Non-Coding |
| >7:47696105_47696306_7:48100875_48101076 | Coding | Non-Coding | Non-Coding | Non-Coding |
| >7:6057782_6057982_1:9779030_9779231 | Coding | Non-Coding | Non-Coding | Non-Coding |
| >7:6469305_6469505_1:596982_597182 | Coding | Non-Coding | Non-Coding | Non-Coding |
| >7:8327541_8327742_7:8343258_8343458 | Non-Coding | Non-Coding | Non-Coding | Non-Coding |
| >1:5442949_5443150_1:5199217_5199418 | Coding | Non-Coding | Non-Coding | Non-Coding |
| >7:9050004_9050205_NW_004515657.1:598233_598434 | Coding | Non-Coding | Non-Coding | Non-Coding |
| >7:9050006_9050207_NW_004515657.1:598231_598432 | Coding | Non-Coding | Non-Coding | Non-Coding |
| >7:9050010_9050211_4:16583180_16583380 | Coding | Non-Coding | Non-Coding | Non-Coding |
| >8:10727988_10728189_6:1324883_1325083 | Coding | Non-Coding | Non-Coding | Non-Coding |
| >8:10728636_10728837_6:1324883_1325083 | Coding | Non-Coding | Non-Coding | Non-Coding |
| >8:10887173_10887373_NW_004516575.1:968_1168 | Coding | Non-Coding | Non-Coding | Non-Coding |
| >8:14152790_14152990_7:44465345_44465546 | Coding | Non-Coding | Coding | Coding |
| >8:1433411_1433611_4:42774251_42774451 | Coding | Non-Coding | Non-Coding | Non-Coding |
| >8:14436854_14437054_8:14760890_14761091 | Coding | Non-Coding | Non-Coding | Non-Coding |
| >8:14693107_14693307_1:1944243_1944443 | Coding | Non-Coding | Non-Coding | Non-Coding |
| >8:14984073_14984274_8:15002359_15002560 | Non-Coding | Non-Coding | Non-Coding | Non-Coding |
| >8:14984074_14984275_8:15002358_15002559 | Non-Coding | Non-Coding | Non-Coding | Non-Coding |
| >8:14984075_14984276_8:15002357_15002558 | Non-Coding | Non-Coding | Non-Coding | Non-Coding |
| >8:14986339_14986540_8:15000222_15000423 | Coding | Non-Coding | Non-Coding | Non-Coding |
| >8:15000222_15000423_8:14986339_14986540 | Coding | Non-Coding | Non-Coding | Non-Coding |
| >8:15002186_15002386_8:14983857_14984057 | Coding | Non-Coding | Non-Coding | Non-Coding |
| >8:2587_2788_1:758_958 | Coding | Non-Coding | Non-Coding | Non-Coding |
| >8:618155_618356_4:13765524_13765725 | Coding | Non-Coding | Non-Coding | Non-Coding |
| >8:6990028_6990229_8:2539503_2539703 | Coding | Non-Coding | Non-Coding | Non-Coding |
| >8:7665122_7665323_6:23492615_23492816 | Coding | Non-Coding | Non-Coding | Non-Coding |
| >8:7760246_7760446_3:34452148_34452348 | Coding | Non-Coding | Non-Coding | Non-Coding |
| >8:8011231_8011432_2:1705684_1705884 | Coding | Non-Coding | Non-Coding | Non-Coding |
| >8:957094_957294_8:938146_938347 | Coding | Non-Coding | Coding | Coding |
| >NW_004515686.1:321645_321846_4:40170426_40170627 | Non-Coding | Non-Coding | Non-Coding | Non-Coding |
| >NW_004515716.1:19252_19453_8:14295954_14296154 | Coding | Non-Coding | Non-Coding | Non-Coding |
| >NW_004515735.1:195251_195451_NW_004517768.1:10167_10368 | Coding | Non-Coding | Non-Coding | Non-Coding |
| >1:6125833_6126034_3:14473737_14473938 | Coding | Non-Coding | Non-Coding | Non-Coding |
| >NW_004515837.1:203633_203834_5:44002398_44002598 | Coding | Coding | Non-Coding | Coding |
| >NW_004515843.1:20074_20275_NW_004522746.1:13660_13860 | Coding | Coding | Non-Coding | Coding |
| >NW_004515920.1:43340_43541_2:32140053_32140253 | Coding | Coding | Coding | Coding |
| >NW_004515953.1:37275_37475_1:29617783_29617984 | Coding | Non-Coding | Non-Coding | Non-Coding |
| >NW_004515983.1:21759_21959_6:49218936_49219136 | Non-Coding | Non-Coding | Non-Coding | Non-Coding |
| >NW_004516029.1:234570_234771_3:37683056_37683257 | Coding | Non-Coding | Non-Coding | Non-Coding |
| >NW_004516029.1:234596_234797_3:37682776_37682977 | Non-Coding | Non-Coding | Non-Coding | Non-Coding |
| >NW_004516230.1:128500_128700_1:5843028_5843228 | Coding | Non-Coding | Non-Coding | Non-Coding |
| >NW_004516230.1:128501_128701_1:5843027_5843227 | Coding | Non-Coding | Non-Coding | Non-Coding |
| >NW_004516243.1:13903_14103_3:452767_452967 | Non-Coding | Non-Coding | Non-Coding | Non-Coding |
| >NW_004516243.1:13903_14103_3:453568_453768 | Non-Coding | Non-Coding | Non-Coding | Non-Coding |
| >NW_004516264.1:262579_262780_6:55393819_55394020 | Coding | Non-Coding | Non-Coding | Non-Coding |
| >NW_004516329.1:553199_553399_6:13838822_13839022 | Non-Coding | Non-Coding | Non-Coding | Non-Coding |
| >NW_004516410.1:2936_3136_NW_004516678.1:50284_50485 | Non-Coding | Non-Coding | Non-Coding | Non-Coding |
| >NW_004516410.1:34638_34838_NW_004516313.1:80976_81177 | Non-Coding | Non-Coding | Non-Coding | Non-Coding |
| >1:6781653_6781854_6:58255085_58255286 | Coding | Non-Coding | Non-Coding | Non-Coding |
| >NW_004516627.1:74667_74868_6:41575553_41575754 | Non-Coding | Non-Coding | Non-Coding | Non-Coding |
| >NW_004516695.1:34424_34624_NW_004516047.1:252887_253087 | Coding | Non-Coding | Non-Coding | Non-Coding |
| >NW_004516723.1:109369_109569_1:47888432_47888633 | Coding | Non-Coding | Coding | Coding |
| >NW_004516753.1:210042_210242_7:13481844_13482044 | Coding | Non-Coding | Non-Coding | Non-Coding |
| >NW_004516931.1:10721_10922_7:2361040_2361241 | Coding | Non-Coding | Non-Coding | Non-Coding |
| >NW_004517596.1:1456_1657_5:20744907_20745107 | Coding | Non-Coding | Non-Coding | Non-Coding |
| >NW_004517817.1:17537_17738_NW_004516931.1:10885_11085 | Non-Coding | Non-Coding | Non-Coding | Non-Coding |
| >NW_004517817.1:17537_17738_NW_004516931.1:8991_9191 | Non-Coding | Non-Coding | Non-Coding | Non-Coding |
| >NW_004517947.1:468_669_NW_004516545.1:96722_96922 | Coding | Non-Coding | Non-Coding | Non-Coding |
| >NW_004517947.1:468_669_NW_004516545.1:96726_96926 | Coding | Non-Coding | Non-Coding | Non-Coding |
| >NW_004522059.1:376_577_NW_004517335.1:55533_55733 | Coding | Non-Coding | Non-Coding | Non-Coding |
| >NW_004522111.1:181_382_1:2855194_2855394 | Coding | Non-Coding | Non-Coding | Non-Coding |
| >NW_004522260.1:973_1173_NW_004521997.1:107_308 | Coding | Non-Coding | Non-Coding | Non-Coding |
| >NW_004522260.1:1026_1226_NW_004521997.1:107_308 | Coding | Non-Coding | Non-Coding | Non-Coding |
| >NW_004522636.1:1763_1964_7:241212_241412 | Coding | Non-Coding | Non-Coding | Non-Coding |
| >NW_004522673.1:466_667_5:34488233_34488433 | Coding | Non-Coding | Non-Coding | Non-Coding |
| >NW_004522673.1:470_671_5:34488237_34488437 | Coding | Non-Coding | Non-Coding | Non-Coding |
| >NW_004522681.1:339_540_6:6273741_6273942 | Coding | Non-Coding | Non-Coding | Non-Coding |
| >NW_004522681.1:339_540_6:6552261_6552461 | Coding | Non-Coding | Non-Coding | Non-Coding |
| >NW_004522716.1:5641_5841_2:10218984_10219184 | Coding | Non-Coding | Non-Coding | Non-Coding |
| >NW_004522716.1:5641_5841_2:10218988_10219188 | Coding | Non-Coding | Non-Coding | Non-Coding |
| >NW_004522716.1:5846_6046_2:10218984_10219184 | Non-Coding | Non-Coding | Non-Coding | Non-Coding |
| >NW_004522716.1:5846_6046_2:10218988_10219188 | Non-Coding | Non-Coding | Non-Coding | Non-Coding |
| >NW_004522742.1:425_626_NW_004520126.1:9640_9840 | Coding | Non-Coding | Non-Coding | Non-Coding |
| >1:8839589_8839790_3:26932874_26933074 | Coding | Coding | Non-Coding | Coding |
| >4:141544_141745_2:1494184_1494384 | Coding | Non-Coding | Non-Coding | Non-Coding |
