# Supplementary material for: Molecular and expression analyses indicate the role of fusion transcripts in mediating abiotic stress responses in chickpea
Source: Front Plant Sci. 2025 Oct 31;16:1677098. doi: 10.3389/fpls.2025.1677098 (PMC12615446; doi:10.3389/fpls.2025.1677098)
Supplement: Supplementary Table 6 — List of differentially expressed genes in samples with and without fusion transcripts. [file Table6.docx]

**Table S6.** List of differentially expressed genes in samples with and without fusion transcripts

| **Upregulated gene** | **Mean of expression in fusion-present samples** | **Mean of expression in fusion-absent samples** | **log2FC** | **p-value** |
| --- | --- | --- | --- | --- |
| LOC101509740 | 7398.081298 | 2643.816231 | 1.484178667 | 4.13E-07 |
| LOC101492545 | 3.66483 | 0.7227166667 | 1.437139063 | 0.0002483991149 |
| LOC101496011 | 28554.06962 | 5187.444997 | 2.460372776 | 5.61E-07 |
| LOC101514962 | 1431.322424 | 411.695088 | 1.795208116 | 0.02363725128 |
| LOC101500209 | 174.997749 | 46.11940146 | 1.901163859 | 0.0001200736819 |
| LOC101494819 | 22.6582773 | 3.007461937 | 2.561584303 | 0.006365778699 |
| LOC101515266 | 264.1650085 | 61.33717454 | 2.088725737 | 3.17E-22 |
| LOC105852659 | 736.9576417 | 110.272488 | 2.729441077 | 0.01833845082 |
| LOC101500209 | 174.997749 | 46.11940146 | 1.901163859 | 0.0001200736819 |
| LOC101499935 | 38.961809 | 11.75154443 | 1.6479499 | 0.0006297797958 |
| LOC101506557 | 1918.960164 | 784.4150483 | 1.289549233 | 0.03692413839 |
| LOC101494120 | 2813.172998 | 990.0434577 | 1.505690793 | 2.44E-08 |
| LOC101514962 | 1070.451575 | 67.99057642 | 3.957023524 | 8.08E-05 |
| LOC113784935 | 4.003826471 | 1.332859 | 1.100932646 | 0.0001585074948 |
| LOC101497135 | 28.71239867 | 12.08853061 | 1.182761937 | 0.003381699399 |
| LOC101496011 | 33412.51016 | 14044.94113 | 1.250278255 | 0.0006994829849 |
| LOC101509484 | 27.76881547 | 12.64182273 | 1.0764694 | 8.46E-06 |
| LOC101497453 | 1632.097667 | 661.3150175 | 1.302021598 | 0.002267758647 |
| CHS | 724.879415 | 122.0761967 | 2.560178137 | 0.01329707952 |
| LOC101512439 | 15.15283086 | 4.180223526 | 1.640700769 | 0.01151948738 |
| LOC101498781 | 12.38228467 | 1.58766465 | 2.370601871 | 0.04615802396 |
| LOC101501730 | 637.882324 | 200.6541383 | 1.663667213 | 0.001077756829 |
| LOC101497325 | 10.56435233 | 4.6960029 | 1.021662684 | 0.000333112118 |
| LOC101507543 | 85.63154167 | 22.5948933 | 1.876417744 | 0.01185689242 |

| **Downregulated** | **Mean of expression in fusion-present samples** | **Mean of expression in fusion-absent samples** | **log2FC** | **p-value** |
| --- | --- | --- | --- | --- |
| LOC101498330 | 0.175468 | 11.74928675 | -3.439109369 | 0.04310547415 |
| LOC101508430 | 0.51442 | 3.531045043 | -1.581078464 | 0.01154382613 |
| LOC101504403 | 0.1712765 | 1.976145875 | -1.345363552 | 0.01513630207 |
