# Supplementary material for: Molecular and expression analyses indicate the role of fusion transcripts in mediating abiotic stress responses in chickpea
Source: Front Plant Sci. 2025 Oct 31;16:1677098. doi: 10.3389/fpls.2025.1677098 (PMC12615446; doi:10.3389/fpls.2025.1677098)
Supplement: Supplementary Table 7 — List of fusion transcripts detected in Long-read RNA-Seq data. [file Table7.docx]

**Table S7.** List of fusion transcripts detected in Long-read RNA-Seq data.

| **Gene1** | **Gene2** | **Breakpoint 1** | **Breakpoint 2** | **Start 1** | **End 1** | **Start 2** | **End 2** | **Predicted length of fusion transcript** |
| --- | --- | --- | --- | --- | --- | --- | --- | --- |
| LOC101512687 | LOC101503191 | 23933994 | 5631 | 23933789 | 23933999 | 5635 | 7225 | 1800 |
| LOC101504980 | LOC101509554 | 30744589 | 13555156 | 30744586 | 30744657 | 13554936 | 13555149 | 284 |
| LOC101493912 | LOC101496628 | 31356943 | 39264029 | 31356717 | 31356943 | 39263978 | 39264032 | 280 |
| LOC101493912 | LOC101509484 | 31358189 | 21635029 | 31357711 | 31358189 | 21634854 | 21635030 | 654 |
| LOC101493912 | LOC101509484 | 31358189 | 21635586 | 31357711 | 31358189 | 21635404 | 21635583 | 657 |
| LOC101514962 | LOC101496011 | 3343313 | 14022203 | 3343304 | 3343879 | 14021571 | 14022203 | 1207 |
| LOC101501345 | LOC101500252 | 16533516 | 2538836 | 16533243 | 16533517 | 2538835 | 2539225 | 664 |
| LOC101501466 | LOC101493912 | 31623291 | 31359283 | 31623174 | 31623292 | 31359282 | 31359798 | 634 |
| LOC101506836 | LOC101497325 | 39988535 | 958 | 39988426 | 39988532 | 736 | 949 | 319 |
| LOC101501663 | LOC101500070 | 6965732 | 6838668 | 6965676 | 6965732 | 6836937 | 6838669 | 1788 |
| LOC101488621 | LOC101509617 | 11378175 | 45752819 | 11377984 | 11378175 | 45752822 | 45752927 | 296 |
| LOC105851910 | LOC101493610 | 19020149 | 26295288 | 19020159 | 19020896 | 26295095 | 26295285 | 927 |
| LOC105851910 | LOC101507919 | 19020392 | 43479567 | 19020402 | 19021328 | 43478842 | 43479574 | 1658 |
| LOC101501044 | LOC101500197 | 3793903 | 3765761 | 3793617 | 3793904 | 3765758 | 3765905 | 434 |
| LOC101502456 | LOC101512740 | 46430084 | 36666746 | 46429965 | 46430084 | 36666744 | 36667405 | 780 |
| LOC101490106 | LOC101489323 | 24450273 | 7679368 | 24448689 | 24450276 | 7679109 | 7679370 | 1848 |
| LOC101490106 | LOC101489323 | 24450273 | 7679791 | 24448689 | 24450276 | 7679748 | 7679793 | 1632 |
| LOC101491017 | LOC105851751 | 887192 | 12123321 | 886826 | 887193 | 12123316 | 12123386 | 437 |
| LOC101504067 | LOC113787067 | 11997177 | 15450781 | 11997032 | 11997173 | 15450730 | 15450781 | 192 |
| LOC101508763 | LOC101501416 | 17855071 | 24426206 | 17855071 | 17855235 | 24426095 | 24426209 | 278 |
| LOC113786767 | LOC101490255 | 20844865 | 33764259 | 20844865 | 20845325 | 33764257 | 33764306 | 509 |
| LOC101491250 | LOC101501855 | 30778772 | 7379193 | 30778480 | 30778772 | 7379192 | 7379427 | 527 |
| LOC101503743 | LOC101505762 | 31763840 | 62137 | 31763840 | 31764007 | 61970 | 62131 | 328 |
| LOC101503743 | LOC101505762 | 31763840 | 62269 | 31763840 | 31764007 | 62270 | 62678 | 575 |
| LOC101500660 | LOC101496430 | 34494770 | 5200 | 34494770 | 34495186 | 5071 | 5206 | 551 |
| LOC101515776 | LOC101509332 | 37640967 | 41564486 | 37640967 | 37641118 | 41564486 | 41564634 | 299 |
| LOC101489433 | LOC101489100 | 38267287 | 38249972 | 38267292 | 38267417 | 38249972 | 38250398 | 551 |
| LOC101502569 | LOC101496222 | 41701939 | 41677974 | 41701734 | 41701939 | 41677975 | 41678176 | 406 |
| LOC101504279 | LOC101501389 | 42648080 | 37837157 | 42647912 | 42648080 | 37836239 | 37837157 | 1086 |
| LOC101514374 | LOC113784864 | 48062932 | 11085 | 48062932 | 48063174 | 11094 | 11387 | 535 |
| LOC113787216 | LOC105852223 | 13838946 | 48156966 | 13838946 | 13839022 | 48156689 | 48156962 | 349 |
| LOC101496011 | LOC101504019 | 14021988 | 10698267 | 14021569 | 14021987 | 10698275 | 10698352 | 495 |
| LOC101496011 | LOC101504019 | 14022099 | 10698267 | 14021615 | 14022100 | 10698275 | 10698352 | 562 |
| LOC101496011 | LOC101514962 | 14022212 | 3343305 | 14021571 | 14022203 | 3343304 | 3343879 | 1207 |
| LOC101496011 | LOC101504019 | 14022288 | 10698275 | 14021731 | 14022278 | 10698275 | 10698352 | 624 |
| LOC113787067 | LOC101504067 | 15450536 | 11997177 | 15450257 | 15450536 | 11997032 | 11997173 | 420 |
| LOC101500131 | LOC101505021 | 20505694 | 673 | 20505612 | 20505695 | 602 | 683 | 549 |
| LOC101502579 | LOC101497514 | 28685347 | 364000 | 28685352 | 28685406 | 363623 | 364002 | 433 |
| LOC101502579 | LOC113784935 | 28685347 | 141098 | 28685352 | 28685406 | 140877 | 141100 | 277 |
| LOC101496683 | LOC105852351 | 34235216 | 34394896 | 34235221 | 34235630 | 34392600 | 34394895 | 2704 |
| LOC101509648 | LOC101512439 | 41576320 | 55393820 | 41576239 | 41576315 | 55393818 | 55393871 | 129 |
| LOC101509648 | LOC101512439 | 41576482 | 55393820 | 41576419 | 41576482 | 55393818 | 55393871 | 116 |
| LOC101509648 | LOC101512439 | 41576635 | 55393513 | 41576100 | 41576633 | 55393507 | 55393893 | 919 |
| LOC101515041 | LOC101508763 | 53304323 | 17861724 | 53304094 | 53304325 | 17861534 | 17861724 | 421 |
| LOC101501310 | LOC101514374 | 53857641 | 48063594 | 53857352 | 53857641 | 48063295 | 48063595 | 589 |
| LOC101513088 | LOC101512439 | 55412531 | 55393820 | 55412458 | 55412533 | 55393818 | 55393871 | 128 |
| LOC101513088 | LOC101512439 | 55412531 | 55393975 | 55412458 | 55412533 | 55393975 | 55394208 | 308 |
| LOC101506890 | LOC101506557 | 58264602 | 58254689 | 58264434 | 58264600 | 58254687 | 58254838 | 317 |
| LOC101505566 | LOC101511040 | 7429102 | 35187921 | 7428393 | 7429103 | 35187921 | 35188155 | 944 |
| LOC101512656 | LOC105851297 | 7603273 | 50285 | 7602928 | 7603273 | 50284 | 50457 | 518 |
| LOC101504938 | LOC101515578 | 9405675 | 5623050 | 9405500 | 9405683 | 5622860 | 5623044 | 367 |
| LOC101509445 | LOC101509981 | 1245565 | 1246950 | 1245567 | 1245643 | 1246946 | 1248204 | 1334 |
| LOC101509445 | LOC101509981 | 1245565 | 1247011 | 1245567 | 1245643 | 1247011 | 1247377 | 442 |
| LOC101500028 | LOC105851688 | 45244313 | 4797206 | 45244307 | 45244575 | 4796914 | 4797202 | 556 |
| LOC101506473 | LOC101495229 | 46720294 | 732 | 46720296 | 46720568 | 733 | 1131 | 670 |
| LOC105852505 | LOC101498120 | 17922471 | 17885841 | 17922290 | 17922469 | 17885841 | 17886071 | 409 |
| LOC101494954 | LOC101498567 | 23896324 | 3361909 | 23896327 | 23898316 | 3361583 | 3361911 | 2317 |
| LOC113787737 | LOC101502451 | 27323257 | 40096167 | 27322398 | 27323256 | 40096165 | 40096739 | 1432 |
| LOC101509032 | LOC101495642 | 28703435 | 596140 | 28703343 | 28703433 | 595481 | 596147 | 756 |
| LOC101501306 | LOC101494709 | 5442950 | 5199218 | 5442942 | 5443011 | 5199224 | 5199624 | 469 |
| LOC101493600 | LOC101490946 | 10728637 | 1325083 | 10728635 | 10728944 | 1325031 | 1325081 | 359 |
| LOC113783912 | LOC113784679 | 10887373 | 1168 | 10886531 | 10887375 | 893 | 1171 | 1122 |
| LOC113788070 | LOC101497325 | 2588 | 958 | 2591 | 2696 | 736 | 949 | 318 |
| LOC105852659 | CHS | 957294 | 938147 | 957102 | 957296 | 938147 | 938277 | 324 |
| LOC101488602 | LOC101510937 | 203634 | 44002598 | 203634 | 203786 | 44002402 | 44002598 | 348 |
| LOC101503481 | LOC101494793 | 20075 | 13860 | 20071 | 20248 | 13851 | 13953 | 279 |
| LOC101490575 | LOC101489157 | 43341 | 32140253 | 43349 | 43902 | 32139508 | 32140255 | 1300 |
| LOC101507060 | LOC101514175 | 128700 | 5843228 | 128707 | 128906 | 5843219 | 5844286 | 1266 |
| LOC101507060 | LOC101514175 | 128701 | 5843227 | 128707 | 128906 | 5843219 | 5844286 | 1266 |
| LOC113784532 | LOC101512439 | 262580 | 55393820 | 262582 | 262672 | 55393818 | 55393871 | 143 |
| LOC101514245 | LOC113787216 | 553399 | 13839022 | 553294 | 553405 | 13838946 | 13839022 | 187 |
| LOC101490268 | LOC105851297 | 3136 | 50285 | 2903 | 3132 | 49049 | 50282 | 1462 |
| LOC113784622 | LOC101492297 | 34838 | 80977 | 34635 | 34846 | 80978 | 81252 | 485 |
| LOC101491443 | LOC101509648 | 74668 | 41575554 | 74663 | 74773 | 41575557 | 41576106 | 659 |
| LOC101506617 | LOC101502632 | 34624 | 253087 | 34560 | 34624 | 252883 | 253087 | 268 |
| LOC113784864 | LOC101498781 | 10722 | 2361041 | 9619 | 10731 | 2360463 | 2361051 | 1700 |
| LOC101506738 | LOC101510273 | 1457 | 20745107 | 1256 | 1464 | 20745030 | 20745104 | 282 |
| LOC101505873 | LOC113784864 | 17538 | 11085 | 17065 | 17542 | 11094 | 11387 | 770 |
| LOC101505873 | LOC113784864 | 17538 | 9191 | 17065 | 17542 | 9195 | 9442 | 724 |
| LOC101489294 | LOC101494367 | 469 | 96922 | 181 | 473 | 96868 | 96931 | 355 |
| LOC101489294 | LOC101494367 | 469 | 96926 | 181 | 473 | 96868 | 96931 | 355 |
| LOC105851490 | LOC101500331 | 377 | 55733 | 373 | 1007 | 55393 | 55742 | 983 |
| LOC101514580 | LOC101512435 | 182 | 2855394 | 181 | 508 | 2854902 | 2855396 | 821 |
| LOC101495229 | LOC101508351 | 1173 | 108 | 1168 | 1376 | 99 | 179 | 288 |
| LOC101495229 | LOC101508351 | 1226 | 108 | 1233 | 1441 | 99 | 179 | 288 |
| LOC101496101 | LOC101510715 | 467 | 34488433 | 181 | 473 | 34488265 | 34488437 | 464 |
| LOC101496101 | LOC101510715 | 471 | 34488437 | 181 | 473 | 34488265 | 34488437 | 464 |
| LOC101498403 | LOC101515780 | 340 | 6273742 | 342 | 507 | 6273752 | 6273818 | 231 |
| LOC101498403 | LOC101495245 | 340 | 6552461 | 342 | 507 | 6552385 | 6552451 | 231 |
| LOC101509074 | LOC101508407 | 5841 | 10219184 | 5656 | 5849 | 10215007 | 10219189 | 4375 |
| LOC101509074 | LOC101508407 | 5841 | 10219188 | 5656 | 5849 | 10215007 | 10219189 | 4375 |
| LOC101509074 | LOC101508407 | 6046 | 10219184 | 5955 | 6046 | 10215007 | 10219189 | 4273 |
| LOC101509074 | LOC101508407 | 6046 | 10219188 | 5955 | 6046 | 10215007 | 10219189 | 4273 |
| LOC101492545 | LOC101510150 | 426 | 9840 | 435 | 552 | 9832 | 10590 | 875 |
| LOC101496413 | LOC101497135 | 141545 | 1494384 | 141545 | 142080 | 1493866 | 1494384 | 1053 |
