# Supplementary material for: Molecular and expression analyses indicate the role of fusion transcripts in mediating abiotic stress responses in chickpea
Source: Front Plant Sci. 2025 Oct 31;16:1677098. doi: 10.3389/fpls.2025.1677098 (PMC12615446; doi:10.3389/fpls.2025.1677098)
Supplement: Supplementary Table 8 — List of RNA-Seq samples used to study intra-specific diversity of fusion transcripts. [file Table8.docx]

**Table S8.** List of RNA-Seq samples used to study intra-specific diversity of fusion transcripts.

| **SRR ID** | **Chickpea genotype** |
| --- | --- |
| SRR12081280 | JG11 |
| SRR12081281 | JG11 |
| SRR12081282 | KAK2 |
| SRR12081283 | KAK2 |
| SRR12081284 | JG11 |
| SRR12081287 | KAK2 |
| SRR12081288 | KAK2 |
| SRR12081289 | KAK2 |
| SRR12081290 | JG11 |
| SRR12081291 | JG11 |
| SRR1298983 | JG62 |
| SRR1298984 | JG62 |
| SRR1298985 | WR315 |
| SRR1298986 | WR315 |
| SRR2079844 | ICC1882 |
| SRR2079847 | ICC1882 |
| SRR2079852 | ICC1882 |
| SRR2079853 | ICC1882 |
| SRR2079854 | ICC1882 |
| SRR2079855 | ICC1882 |
| SRR2183533 | WR315 |
| SRR2183534 | K850 |
| SRR2183535 | JG62 |
| SRR3313137 | JGK3 |
| SRR3313138 | JGK3 |
| SRR3313139 | JGK3 |
| SRR3313140 | JGK3 |
| SRR3313141 | JGK3 |
| SRR3313142 | JGK3 |
| SRR3313143 | JGK3 |
| SRR3313144 | JGK3 |
| SRR3313145 | JGK3 |
| SRR3313146 | JGK3 |
| SRR3313150 | JGK3 |
| SRR3313151 | JGK3 |
| SRR3313152 | JGK3 |
| SRR3313153 | JGK3 |
| SRR3313154 | JGK3 |
| SRR3313155 | JGK3 |
| SRR3313156 | JGK3 |
| SRR3313157 | JGK3 |
| SRR3313158 | JGK3 |
| SRR3313159 | JGK3 |
| SRR3313160 | JGK3 |
| SRR3313161 | Himchana 1 |
| SRR3313162 | Himchana 1 |
| SRR3313163 | Himchana 1 |
| SRR3313164 | Himchana 1 |
| SRR3313165 | Himchana 1 |
| SRR3313166 | Himchana 1 |
| SRR3313167 | Himchana 1 |
| SRR3313168 | Himchana 1 |
| SRR3313169 | Himchana 1 |
| SRR3313170 | Himchana 1 |
| SRR3313171 | Himchana 1 |
| SRR3313172 | Himchana 1 |
| SRR3313173 | Himchana 1 |
| SRR3313174 | Himchana 1 |
| SRR3313176 | Himchana 1 |
| SRR3313177 | Himchana 1 |
| SRR3313178 | Himchana 1 |
| SRR3313179 | Himchana 1 |
| SRR3313180 | Himchana 1 |
| SRR3313181 | Himchana 1 |
| SRR3313182 | Himchana 1 |
| SRR3313183 | Himchana 1 |
| SRR3313184 | Himchana 1 |
| SRR3990783 | BG362 |
| SRR3990784 | P256 |
| SRR3990786 | P256 |
| SRR3990788 | BG362 |
| SRR5435262 | JG62 |
| SRR5435263 | JG62 |
| SRR5435264 | JG62 |
| SRR5435265 | JG62 |
| SRR5435266 | JG62 |
| SRR5435267 | JG62 |
| SRR5927129 | Hashem |
| SRR5927130 | Hashem |
| SRR5927131 | Hashem |
| SRR5927132 | Hashem |
| SRR5927133 | Bivanij |
| SRR5927134 | Bivanij |
| SRR5927135 | Bivanij |
| SRR5927136 | Bivanij |
| SRR7686346 | Pb7 |
| SRR7686347 | C214 |
| SRR7686348 | ICCV 05530 |
| SRR7686349 | BC3F6 |
| SRR7686351 | C214 |
| SRR7686352 | C214 |
| SRR7686353 | ICCV 05530 |
| SRR7686354 | Pb7 |
| SRR7686355 | C214 |
| SRR7686356 | ILC 3279 |
| SRR7686357 | Pb7 |
| SRR7686359 | BC3F6 |
| SRR7686360 | ILC 3279 |
| SRR7686361 | Pb7 |
| SRR7686362 | ICCV 05530 |
| SRR7686363 | ICCV 05530 |
| SRR7686364 | ILC 3279 |
| SRR7686365 | ILC 3279 |
