# Supplementary material for: Molecular and expression analyses indicate the role of fusion transcripts in mediating abiotic stress responses in chickpea
Source: Front Plant Sci. 2025 Oct 31;16:1677098. doi: 10.3389/fpls.2025.1677098 (PMC12615446; doi:10.3389/fpls.2025.1677098)
Supplement: Supplementary Table 9 — List of primers used for qRT-PCR of fusion transcripts and their parental genes. [file Table9.docx]

**Table S9.** List of primers used for qRT-PCR of fusion transcripts and their parental genes.

| **Fusion ID** | **Forward primer** | **Reverse primer** |
| --- | --- | --- |
| LOC101498330_LOC113785383 | CTGGAGAGAATTGGACCTGC | AGGCTTCACGTGGTTAGA |
| LOC101489433_LOC101489100 | GATGTTGATTCTGACGGG | GACTGTTGCGAATGAGAGC |
| LOC101506206_LOC101493600 | GCTTGAATTGGAGGAGGCG | GAAGTGAAAAGCCCGGTGC |
| LOC101515613_LOC113787786 | CGGGTTATCAGAATCAGC | CAATGCCTTCACATCCC |
| LOC101494819_LOC101493433 | TGGTGGCAAACAAGGCTGAT | CAATTCGAGGTCTTCTTGTC |
| LOC101506473_LOC101495229 | CCTGAAATCCCCCCTTTCT | AGGAGTTTCATTGGCACAGC |
| LOC101495229_LOC101508351 | GCCTTGATCTTGGAGGAGAA | GTTTTGGGACATTTGCTCG |
| LOC101500131_LOC101505021 | AGCTACCATAAGCCAGCC | GCATAATCGGAGAGAAGTTG |
| LOC101509445_LOC101509981 | TCTCTCCCTCCTCCAGAT | CTGTGGCTCTTCTGCAATTA |
| **Fusion Parental gene** | **Forward primer** | **Reverse primer** |
| LOC101500131 | CGGAGAGAGCAGCCTACA | GCAGACCTGAATTGGAGGC |
| LOC101505021 | GGGCCTTCTCTGTTCTATG | GACTGAAGCTTGCGGGGTGT |
| LOC101495229 | ATGGAGATCAACCACAATGCC | GGATAAGGACCATCAAGAAGG |
| LOC101508351 | GAGGGCTTTGCTTTTTCTCAC | CTCCCAAGAAAGCAACAACC |
| LOC101506206 | GCTTGAATTGGAGGAGGCGAT | GCACGAACTTTCAACATGGC |
| LOC101493600 | GAGTGGAAGGGAGTGGGATA | GAGGAGAAGTGAAAAGCCCG |
| EF1*α* | TCCACCACTTGGTCGTTTTG | CTTAATGACACCGACAGCAACAG |
